# Antifungal Agents’ Trends of Utilization, Spending, and Prices in the US Medicaid Programs: 2009–2023

**DOI:** 10.3390/antibiotics14050518

**Published:** 2025-05-16

**Authors:** Abdulrahman A. Alsuhibani, Norah A. Alobaid, Manar H. Alahmadi, Jood S. Alqannas, Wejdan S. Alfreaj, Rana F. Albadrani, Khalid A. Alamer, Yasser S. Almogbel, Ali Alhomaidan, Jeff J. Guo

**Affiliations:** 1 Department of Pharmacy Practice, College of Pharmacy, Qassim University, Buraydah 51452, Saudi Arabia; norah.abdulaziz.alobaid@gmail.com (N.A.A.); manar.husseen@gmail.com (M.H.A.); joodqanas@gmail.com (J.S.A.); wejdanalfreaj@gmail.com (W.S.A.); ranafalbadrani@gmail.com (R.F.A.); y.almogbel@qu.edu.sa (Y.S.A.); 2 Pharmacy Practice Department, College of Pharmacy, Imam Abdulrahman Bin Faisal University, Dammam 31441, Saudi Arabia; kaalamer@iau.edu.sa; 3 The Saudi Food and Drug Authority, Riyadh 13513, Saudi Arabia; ahomaidan@sfda.gov.sa; 4 James L. Winkle College of Pharmacy, University of Cincinnati Academic Health Center, Cincinnati, OH 45267, USA; guoje@ucmail.uc.edu

**Keywords:** antifungal medication, utilization, spending, trends, COVID-19, Medicaid

## Abstract

**Background:** Fungal infections, particularly among immunocompromised individuals, present significant challenges due to rising incidence rates, treatment costs, and increasing resistance to antifungal agents. This study evaluates trends in antifungal use among Medicaid beneficiaries, focusing on prescribing patterns, costs, and pricing to optimize therapy. **Methods:** Using the national Medicaid outpatient pharmacy claims data collected by the US Center of Medicare and Medicaid Services, a retrospective drug utilization analysis was conducted for antifungal medications from 2009 to 2023. Antifungal medications were categorized based on therapeutic use. The study examined annual utilization, reimbursement, and pricing trends, along with the market share. **Results:** Overall Medicaid utilization of superficial fungal infections’ (SFIs’) medications increased from 3.95 million prescriptions in 2009 to 6.16 million in 2023. Nystatin was the most frequently utilized SFI agent, while fluconazole emerged as the most commonly prescribed agent for invasive fungal infections (IFIs). In 2022, a notable spike occurred in the number of prescriptions for both SFIs and IFIs. Medicaid’s total expenditure on SFI medications rose from USD 121.9 million in 2009 to USD 155 million in 2023, while spending on IFI medications fluctuated substantially, peaking at USD 156.8 million in 2022 before declining to USD 80.7 million in 2023. After being introduced to the market, efinaconazole became the most expensive SFI agent over the years. Isavuconazole, the latest approved IFI medication, demonstrated sustained utilization, reimbursement, and price increases. **Conclusions:** The substantial rise in antifungal utilization and spending underscores the growing financial burden on Medicaid, emphasizing the need for policy interventions to manage costs and generic drug substitution while ensuring equitable access to these essential treatments. However, this study is limited by the lack of clinical outcome data and information on off-label use. Additionally, reimbursement data may not accurately reflect actual drug prices.

## 1. Introduction

Fungal infections present a significant challenge in clinical settings, leading to considerable healthcare expenses and notable rises in morbidity and mortality rates, particularly among at-risk patients [1]. Fungal infections are broadly classified into two primary categories: superficial fungal infections (SFIs) and invasive fungal infections (IFIs). SFIs, including conditions such as tinea pedis, sporotrichosis, and vulvovaginal candidiasis, primarily impact the skin, hair, nails, genitalia, and mucosal surfaces. In contrast, IFIs, such as aspergillosis, candidiasis, histoplasmosis, and cryptococcosis, pose significant threats to vital organs such as the heart, lungs, and brain [2]. The severity of IFIs is underscored by their ability to invade normally sterile body areas, such as the bloodstream, liver, kidneys, lungs, and central nervous system, which contributes to their higher mortality rates [3].

The incidence of fungal infections has markedly increased, particularly among immunocompromised individuals, such as those with cancer, acquired immunodeficiency syndrome (AIDS), and organ transplant recipients. Furthermore, there is a growing prevalence of fungal infections in patients with sepsis. Antifungal agents remain the primary treatment modality for these infections, administered through various routes. Recent advancements have led to the development of several antifungal agents to address the rising incidence and associated high mortality rates [4]. The history of antifungal drugs began in the 1950s with the introduction of polyenes such as nystatin, natamycin, and amphotericin B-deoxycholate, with the last becoming a cornerstone in treating systemic fungal infections despite its severe side effects, such as infusion-related toxicity and nephrotoxicity, which later drove the development of other antifungal drug classes [5].

Antifungal medications are categorized into many classes to treat different types of infections based on their modes of action. Polyenes such as amphotericin B break down fungal cell membranes, azoles like fluconazole prevent fungal cells from synthesizing ergosterol, and echinocandins prevent the synthesis of cell walls. Each class of antifungals can treat both superficial and systemic mycoses by targeting distinct fungal species and is designed to treat certain fungal diseases [6].

The growing incidence of fungal infections globally is anticipated to propel the global market for antifungal medications to USD 20.52 billion by 2030. Fungal infections have also become more common in recent years due to the expanding use of immunosuppressive and antineoplastic drugs, prosthetic devices, grafts, and broad-spectrum antibiotics [7]. Therefore, the global consumption of systemic antifungal agents between 2008 and 2018 increased from 0.50 to 0.92 defined daily dose (DDD)/1000 inhabitants/day [4]. In recognition of the growing threat, the World Health Organization (WHO) released the ‘WHO Fungal Priority Pathogens List to Guide Research, Development, and Public Health Action’ in 2022, identifying key fungal pathogens that pose major public health concerns [8].

In the United States (U.S.), few studies assessed antifungal consumption. A study analyzing Medicaid data from 1991 to 2009 showed that the use of antifungal agents for IFIs increased nearly tenfold during the study period, with branded fluconazole being the leading drug. In comparison, prescriptions for SFIs saw at least a fourfold increase, with azoles dominating the market, followed by polyenes, particularly nystatin [2]. According to another US study, antifungal medication prices increased significantly between 2000 and 2019. The costs of 139 antifungal medication products, which contain 27 different active ingredients, increased by 50% on average, with 20% of these medications experiencing a 25% price increase [9].

Antifungal resistance (AFR) remains critically underexplored, with key gaps in antifungal stewardship (AFS) programs, particularly in low- and middle-income countries. Limited research, poor diagnostic tools, and inadequate educational initiatives aimed at AFR have led to suboptimal prescribing practices and insufficient management strategies. Addressing these gaps is essential to combat the rising threat of AFR and improve antifungal use [6,10].

The present study aimed to evaluate the trends in utilization, expenditure, and pricing of antifungal agents prescribed for IFIs and SFIs among Medicaid beneficiaries. It provides comprehensive insights to support payers and healthcare providers in balancing cost concerns, safety profiles, and therapeutic efficacy in antifungal therapy selection.

## 2. Results

### 2.1. Utilization

**Figure 1:**
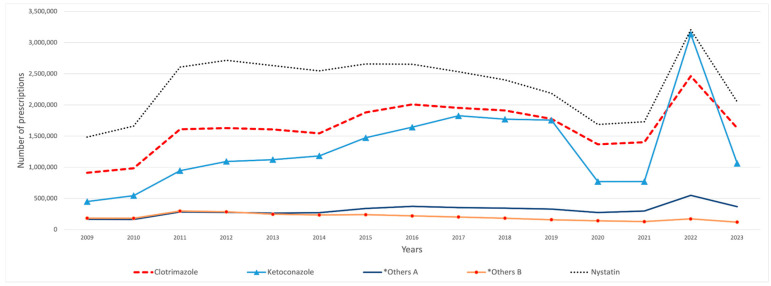
SFI medications’ utilization (number of prescriptions) from 2009 to 2023. Antifungal medications with fewer than 100,000 prescriptions were removed from the graph. *Others A include: terconazole, terbinafine, and ciclopirox. *Others B include: griseofulvin and miconazole.

The overall Medicaid utilization of SFI medications increased from 3.95 million prescriptions in 2009 to 6.16 million in 2023. During the study period, nystatin was the most frequently utilized SFI agent, with a total of 34.75 million prescriptions, followed by clotrimazole with 24.67 million and ketoconazole with 19.55 million, while tioconazole had the lowest number of prescriptions, with only 932 prescriptions. Butoconazole exhibited a significant decline in utilization, with a 98% decrease by 2010. This decline continued until 2013, when utilization suddenly increased by 24,890%. In 2011, a notable rise in the overall utilization of SFI medications was observed, surging by 62% from 4.2 million to 6.9 million (Table 1). Most of this growth was driven by nystatin, which accounted for approximately one million additional prescriptions, followed by clotrimazole, which increased by about 660,000 prescriptions, and ketoconazole, which experienced a rise of around 400,000 prescriptions (Figure 1). In 2012, the utilization of oxiconazole and naftifine significantly decreased by 74% and 69%, respectively, with only 19,082 prescriptions for oxiconazole and 15,177 for naftifine in that year. This downward trend persisted until 2022, when utilization subsequently increased. After efinaconazole appeared on the market in 2014, its utilization experienced significant growth over the years, increasing by 9761% from 2014 to 2023 (Table 1).

**Figure 2:**
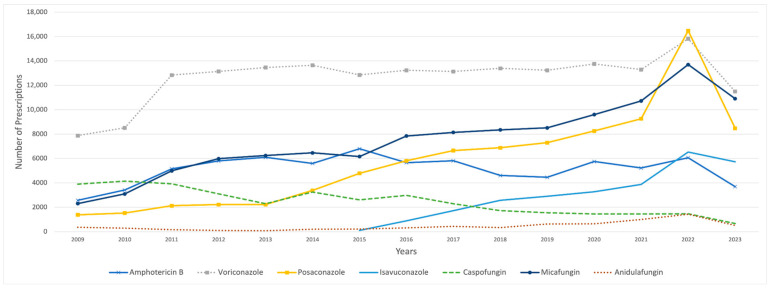
Utilization of IFI medications (number of prescriptions) from 2009 to 2023. Fluconazole was excluded due to its outlier prescription volume, which skewed the graph’s scale. Similarly, itraconazole was removed because of a sharp decline in prescriptions in 2010, which distorted the visual representation.

On the other hand, the majority of IFI medications, with the exceptions of fluconazole and itraconazole, displayed sustained fluctuations throughout the study period (Figure 2). Fluconazole emerged as the most frequently utilized IFI agent, accounting for a total of 37.22 million prescriptions, followed by itraconazole with 560,961 prescriptions and voriconazole with 189,683 prescriptions, while flucytosine had the fewest prescriptions, totaling 1531 prescriptions during the study period. Itraconazole prescriptions significantly declined from 215,261 in 2009 to 27,047 in 2010, a decrease counterbalanced by a notable rise in fluconazole prescriptions. In 2011, similar to the trend observed with SFI medications, total prescriptions for IFI medications demonstrated a substantial increase of 57%, growing from 1.4 million to 2.2 million prescriptions. This increase was mainly attributed to fluconazole, which accounted for an increase of approximately 800,000 prescriptions. Since its introduction to the market, isavuconazole has demonstrated consistent growth in utilization, increasing from 102 prescriptions in 2015 to 5732 prescriptions in 2023 (Table 2).

In 2020, the number of prescribed SFI medications declined by approximately 30%, decreasing from 7.1 million to 5 million. Meanwhile, the total prescriptions for IFI medications decreased by 66%. This decline was primarily attributed to fluconazole, which saw a reduction from 3.2 million to 1 million prescriptions. In contrast, there was a notable spike in the number of prescribed SFIs and IFIs in 2022, rising by 106%, from 5.11 million to 10.56 million, and by 362%, from 1.2 million to 5.6 million prescriptions, respectively. That year, fluconazole, in particular, showed an approximately fourfold rise in the number of prescriptions (Table 1 and Table 2).

### 2.2. Reimbursement

Medicaid’s total expenditure on SFI medications rose from USD 122 million in 2009 to around USD 155 million in 2023. In 2010, there was a reduction of USD 10 million in total spending on SFI medications. This trend was reversed in the subsequent year, leading to a notable 67.56% expenditure increase. Notably, butoconazole experienced a significant decline of 98% in 2010, decreasing from USD 1.14 million to USD 23,471, followed by a remarkable increase of 31,584% in 2013. After that year, total reimbursement costs on SFI medications fluctuated until 2022, when a sharp increase of approximately USD 120 million was observed (Table 3).

**Figure 3:**
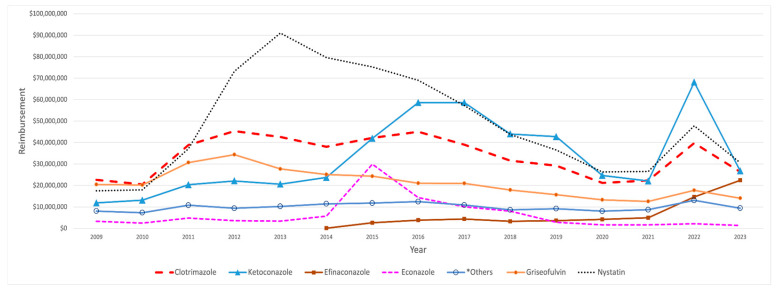
CMS spending for SFI medications from 2009 to 2023. The graph highlights key medications, excluding those with minimal spending. *Others include: terconazole and ciclopirox.

Medicaid’s highest expenditure on SFI medication was for nystatin, which peaked at USD 91 million in 2013 and subsequently experienced a continuous decline, eventually dropping to around USD 31 million by 2023, aside from a notable spike observed in 2022. On the other hand, ketoconazole experienced a more gradual initial increase that continued to 2014, after which it showed a noteworthy jump, hitting around USD 60 million in 2016 and 2017, followed by a steady decline until 2022. That year, spending surged, peaking at around USD 70 million. Econazole maintained consistently low spending levels throughout the years. However, it experienced a sharp spike in 2015, reaching USD 30 million before declining and stabilizing at minimal levels again (Figure 3). Efinaconazole, launched in 2014 with an initial reimbursement cost of USD 92,298, saw its spending soar by 2746% by 2015. Over the entire period from 2014 to 2023, its total reimbursement costs increased dramatically by 24,218%, reaching USD 22.45 million, with a notable spike in 2022, when costs rose from USD 5 million to USD 14.7 million (Table 3).

**Figure 4:**
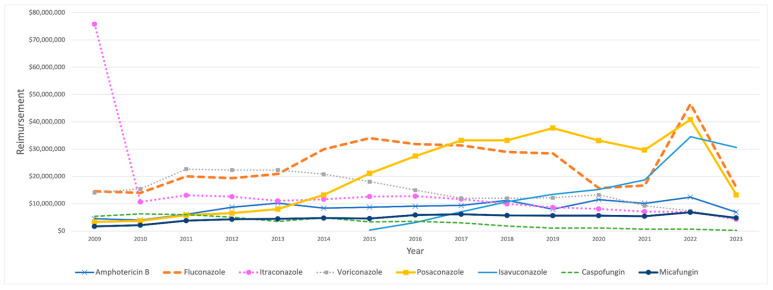
CMS spending for IFI medications from 2009 to 2023. The graph excludes anidulafungin and flucytosine.

On the other hand, Medicaid’s total spending on IFI medications exhibited notable fluctuations over the study period, peaking at USD 156.8 million in 2022 before declining to USD 80.7 million in 2023. In 2009, the most significant portion of Medicaid’s expenditure on IFI medications was attributed to itraconazole, amounting to USD 75.78 million. However, its reimbursement costs dropped sharply, by 86%, in the subsequent year, falling to USD 10.65 million. This marked decrease significantly impacted overall spending for IFI medications, which decreased from USD 119.64 million to USD 56.37 million. This decline in reimbursement was gradually reversed throughout the study period. Isavuconazole’s reimbursement amount began at USD 347,268 in 2015. It showed a large jump in the following year, reaching USD 3.1 million, followed by steady growth over the years until 2022, when a significant 84% increase occurred, rising from USD 18.7 million to USD 34.6 million (Table 4). In contrast, caspofungin reimbursement costs steadily declined throughout the study period (Figure 4).

### 2.3. Prices

**Figure 5:**
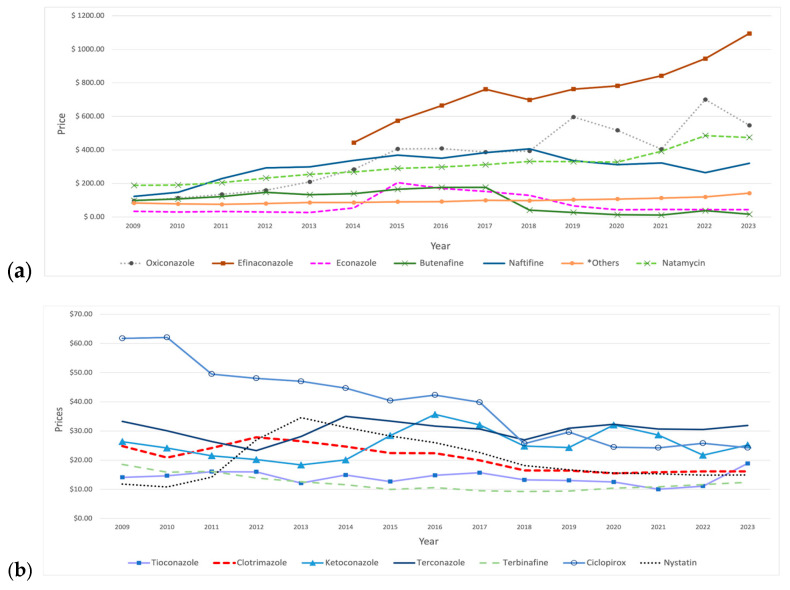
Prices (reimbursement per prescription) for SFI medications from 2009 to 2023. (**a**) The graph includes medications priced above USD 60. *Others include: griseofulvin and butoconazole. (**b**) The graph includes medications priced below USD 60.

Among SFI medications, oxiconazole, efinaconazole, and naftifine were the most expensive agents throughout the study period. Oxiconazole had a pronounced growth, showing fluctuations after 2015, with an increase of 470% from 2009 to 2023, as its price rose from USD 96 to USD 547. Moreover, naftifine and natamycin experienced steady price increases over the years, with naftifine’s growth stopping in 2018 before it began to decline. Upon its introduction to the market in 2014, efinaconazole achieved the highest price, of USD 443, compared to other SFI agents, exhibiting a consistent upward trend, except for a slight decline observed in 2018. In contrast, clotrimazole and ciclopirox experienced sustained price reductions during the study period, with clotrimazole peaking in 2012. Furthermore, miconazole experienced a sharp decline initially, which persisted until 2012, after which it exhibited minor fluctuations over time, including a slight increase in 2022. That year, there was a price increase for most SFI medications, followed by a subsequent decline in the following year (Figure 5).

Price fluctuations were observed for certain medications. For example, econazole showed a significant increase in 2015, followed by a continuous decline in the subsequent years. Butenafine’s price had sustained growth over the years until 2018, when a significant 77% price reduction occurred, followed by a gradual decline with a slight increase in 2022. Additionally, nystatin demonstrated price growth in the initial years, reaching a peak of USD 35 in 2013, followed by a decline in price in the subsequent years. Moreover, ketoconazole’s price initially showed a steady decline until 2013, then reversed and rose, reaching a peak of USD 36 in 2016, followed by fluctuations in the subsequent years. Other medications did not show pronounced price changes (Table 5).

**Figure 6:**
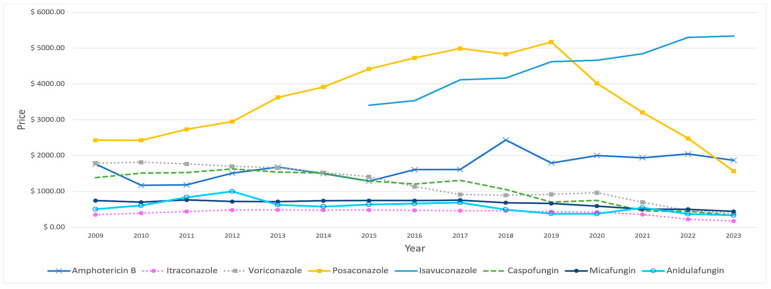
Prices (reimbursement per prescription) for IFI medications from 2009 to 2023, excluding fluconazole and flucytosine.

Among IFI medications, flucytosine had the highest average price, of USD 5789, over the study period. Posaconazole was initially the second most expensive choice, with a substantial price increase over the years. However, this upward trend reversed in 2020, when its price dropped and isavuconazole’s price growth overshadowed it (Figure 6). In comparison, fluconazole stands out as the lowest-priced IFI drug, with an average price of USD 10.63. Amphotericin B demonstrated constant price fluctuations over the years, with a notable spike observed in 2018. Conversely, both caspofungin and voriconazole have consistently experienced a downward price trend over time. In contrast, the other IFI medications’ prices showed no considerable changes over the years, with the exception of anidulafungin, which reached USD 1000 in 2012 (Table 6).

### 2.4. Market Share

The market share of antifungals for SFIs corresponds closely to the previously discussed aspects of utilization and reimbursement. Nystatin maintained the highest market share with an average of 34.91%, followed by clotrimazole at 24.58% and ketoconazole at 18.38%. The utilization markets for nystatin and clotrimazole were relatively stable over the study period. In contrast, ketoconazole demonstrated sustained growth over the years, which ceased in 2020, leading to a subsequent decline before reaching a peak in 2022 (Figure S1, Supplementary Materials). Regarding reimbursement, nystatin also led with the highest market share, averaging 25.71%, with a peak of 42.54% in 2013. Clotrimazole and ketoconazole followed, with average market shares of 18.63% and 18.08%, respectively. Efinaconazole demonstrated remarkable growth, rising from 0.05% in 2014 to 14.49% in 2023. Additionally, econazole showed a notable increase in 2015, jumping from 2.48% to 12.06%, before declining for the rest of the study period (Figure S2, Supplementary Materials).

Among IFI medications, fluconazole was the market frontrunner in prescription rates throughout the study period, achieving an average rate of 96%. Additionally, reimbursement market share trends have shifted over time, reflecting changes in reimbursement policies. Itraconazole was initially dominant in 2009 at over 63.34% of total reimbursement before declining to 18.9% in 2010. This decrease contributed to an increase in the reimbursement share for other IFI medications during that same year. Isavuconazole showed constant growth in reimbursement market share since its introduction, starting from 0.34% in 2015 to 37.9% in 2023 (Figure S3, Supplementary Materials).

### 2.5. Joinpoint Regression

#### 2.5.1. Superficial Fungal Infections’ Medications

##### Utilization

The joinpoint regression revealed that some SFI medications had significant increases in the initial years, which is mainly attributed to the spike that occurred in 2011. Using the 1 Joinpoint model, nystatin, griseofulvin, and clotrimazole exhibited a notable rise in the first four years with an annual percent change (APC) of 24.04%, 22.65%, and 26.21% (*p*-value < 0.05), respectively. After 2012, nystatin and griseofulvin showed a constant and significant decline with an APC = −2.99% and −11.71% (*p*-value < 0.05), respectively, while clotrimazole experienced insignificant fluctuations over the years, which was represented by a flat line with an APC = −0.41% (*p*-value < 0.05). In addition, terconazole, tioconazole, and miconazole also demonstrated marked spikes until 2011, when their APCs = 33.92%, 72.40%, and 20.19% (*p*-value < 0.05), respectively. After that, a steady decrease in the curve was observed (Figure S4, Supplementary Materials).

Using the 2 Joinpoints model, both ketoconazole and terbinafine demonstrated similar patterns; both showed sustained but insignificant growth, with APCs = 17.35% until 2016 and 20.62% (*p*-value < 0.05) until 2017, respectively, when the curve started to move down, with a subsequent increase after 2020. Econazole’s line began with a steady increase, with APC = 4.64% (*p*-value < 0.05) from 2009 to 2015, followed by a decline until the end of the study, with APC = −19.47% (*p*-value < 0.05). Natamycin’s APC significantly rose (APC = 11.06%, *p*-value < 0.05) from 2009 to 2020. Then, its APC significantly dropped (APC = −26.79%, *p*-value < 0.05) for the rest of the study period. For ciclopirox, the APC was continuously growing over the years (APC = 9.53%, *p*-value < 0.05) (Figure S4, Supplementary Materials).

**Figure 7:**
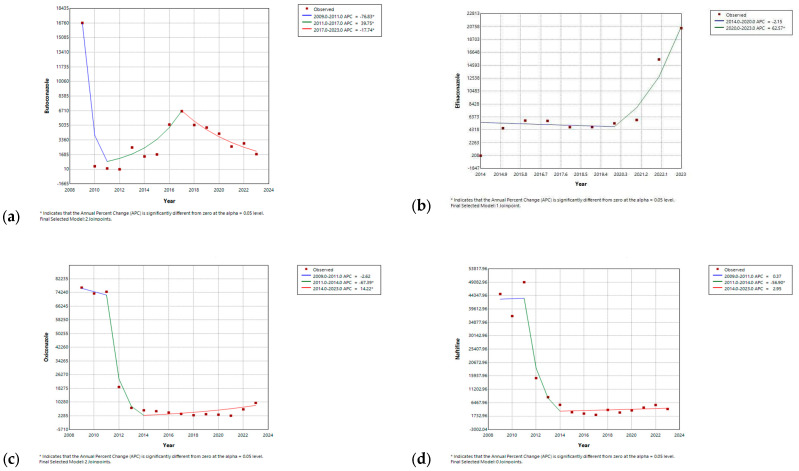
Joinpoint regression for utilization of (**a**) butoconazole; (**b**) efinaconazole; (**c**) oxiconazole; and (**d**) naftifine. * Indicates that the Annual Percent Change (APC) is significantly different from zero at the alpha = 0.05 level.

In contrast, butoconazole initially experienced a significant drop in the first three years (APC = 76.82%, *p*-value < 0.05), with a subsequent significant increase in APC, of 39.74%, until 2017 (*p*-value < 0.05), after which there was a gradual decrease, with an APC of −17.72% (*p*-value < 0.05) until the end of the study (Figure 7). Butenafine also showed a progressive decline, with an APC of −27.78% (*p*-value < 0.05) until 2015, followed by a gradual increase for the remainder of the study period. Efinaconazole initially exhibited a plateaued APC from its market entry until 2020 (APC = −2.09%, *p*-value < 0.05), followed by a significant increase throughout the remainder of the study, with APC reaching 62.5% (*p*-value < 0.05) (Figure S10, Supplementary Materials). Oxiconazole and naftifine displayed comparable trends, initially remaining stable until 2011, followed by marked declines through 2014, with APCs of −67.39% and −56.90% (*p*-value < 0.05), respectively. Subsequently, the trend for both medications stabilized, reaching plateaus (Figure 7).

##### Reimbursement

The reimbursement cost trends for griseofulvin, tioconazole, natamycin, ketoconazole, terbinafine, butoconazole, and efinaconazole closely mirror the patterns observed in their utilization curves, as indicated by joinpoint regression analyses. In contrast, the changes in reimbursement costs for the other medications did not align with the patterns seen in their prescription rates. Nystatin and clotrimazole spending exhibited more pronounced initial growth compared to their utilization curves, with APC = 57.28% and 31.53% (*p*-value < 0.05), followed by a steeper decline (APC = −12.28% and −5.55%, *p*-value < 0.05), respectively, resulting in a narrower angle. In contrast, terconazole showed a more gradual increase over a more extended period of time (APC = 8.85%, *p*-value < 0.05), with a sharper, fluctuated decline (APC = −3.55%, *p*-value < 0.05) when compared with its utilization slope. Over the initial three years, both oxiconazole and naftifine demonstrated an upward trend, with naftifine experiencing a more pronounced increase in 2011. However, both medications significantly declined from 2011 to 2014, characterized by approximately equivalent annual percentage changes (APC). Subsequently, both agents entered a plateau phase. This trend was reversed for oxiconazole after 2021 and saw a substantial increase in APC, reaching 78.29%. Butenafine and miconazole exhibited consistent decreases over the years. However, this downward trend ceased for miconazole in 2015, resulting in a stabilization of its utilization at a steady state. Econazole showed initial fluctuations, followed by a notable peak in 2015, with a subsequent sharp decline. Ciclopirox exhibited no discernible pattern, as its levels fluctuated throughout the study period (Figure S5, Supplementary Materials).

##### Prices

The joinpoint regression analyses demonstrated that the prices of butoconazole, efinaconazole, natamycin, and oxiconazole consistently grew throughout the study period. In contrast, ciclopirox showed a continuous decline. Terbinafine also started with a gradual decline until 2018, after which its price reversed and began to increase until the end of the study period. On the other hand, griseofulvin saw a slight and fluctuating decline until 2016, followed by a gradual escalation through the end of the study. In comparison, clotrimazole began with a slight rise, then shifted to a gradual decline from 2013 to 2023. Butenafine had a more prolonged increase during its early years, continuing until 2017, after which the APC fell, amounting to a decline of −38.86% (*p*-value < 0.05). Naftifine’s initial rise during the first four years was significant, followed by a continued, though less steep, increase until 2018, at which point a gradual decline commenced. Ketoconazole and terconazole both experienced a similar declining trend in the early years, which was reversed by a continuous increase that persisted for three years, beginning in 2013 for ketoconazole and in 2012 for terconazole, before a reduction resumed in the following years. The price trend of nystatin closely reflects its utilization pattern. In contrast, the price curves of econazole and miconazole align with changes in their reimbursements, with econazole showing a more gradual decline after the peak in 2015. On the other hand, tioconazole’s price demonstrated fluctuating changes during the study, with no identified pattern (Figure S6, Supplementary Materials).

#### 2.5.2. Invasive Fungal Infections’ Medications

##### Utilization

One Joinpoint model distinctly illustrates the previously discussed significant drop in itraconazole utilization in 2010, followed by stabilization at a plateau with minimal variation throughout the study period. On the other hand, the APCs of amphotericin B, micafungin, and voriconazole started with significant growth until 2012 (APC = 36.58%, 37.43%, and 22.21% (*p*-value < 0.05), respectively). After that, APC of amphotericin B exhibited a slow decline, whereas that of micafungin continued to grow steadily, albeit at a slower rate, while voriconazole’s remained relatively stable for most years. Fluconazole had a more gradual increase over a more extended period of time before reversing and declining back to baseline levels by the end of the study period. Isavuconazole and posaconazole saw consistent and significant increases over the years, with APCs of 20.83% and 10.96% (*p*-value < 0.05), respectively. In contrast, caspofungin showed a steady decline throughout the study period, with an APC of −8.66%. Anidulafungin began with a gradual decrease until 2013, followed by a significant rise, before increasing in 2023 (Figure S7, Supplementary Materials).

##### Reimbursement

Itraconazole, isavuconazole, caspofungin, and anidulafungin had reimbursement trends similar to their patterns. Amphotericin B exhibited a significant rise until 2012, reversed by growing fluctuations until the study’s conclusion. Similarly, fluconazole demonstrated a growing APC until 2015, after which it started to decline. Furthermore, the reimbursement costs for micafungin and posaconazole continuously rose until 2017, followed by slight, insignificant decreases thereafter. Voriconazole saw a significant initial spike, which was followed by a gradual decline over time (Figure S8, Supplementary Materials).

##### Prices

Amphotericin B exhibited an initial price reduction, followed by an increase in APC starting from 2011 to 2023. In contrast, anidulafungin saw a significant rise until 2012, with an APC of 21.14% (*p*-value < 0.05), reversed by a continuous decline in APC. Caspofungin and micafungin, on the other hand, had relatively more stable prices initially, with subsequent declines after 2014 and 2017, respectively. Itraconazole gradually increased from 2009 to 2012, followed by a slow and minimal decrease until 2020, after which a steeper decline occurred in the subsequent years. Posaconazole experienced sustained price growth, followed by a significant drop from 2019 to the end of the study period. Additionally, isavuconazole showed a consistent price escalation, while voriconazole had a progressive price decline over the study period. Fluconazole, the least costly IFI medication, showed minimal price fluctuations over the years (Figure S9, Supplementary Materials).

## 3. Discussion

### 3.1. Utilization Trends

Over the study period, prescriptions for SFIs increased from 3.95 million in 2009 to 6.16 million in 2023, reflecting the rising burden of fungal infections in outpatient settings across the United States. Consistent with our findings, a 2021 study of Medicare Part D beneficiaries demonstrated a similar increase in antifungal prescriptions [11]. This aligns with global trends, as systemic antifungal use grew by 6.2% annually across 65 countries from 2008–2018, primarily driven by the global burden of superficial fungal infections affecting over one billion people worldwide [4]. Notably, nystatin remained the most commonly prescribed antifungal, with a total of 34.75 million prescriptions recorded. Its continued dominance mirrors earlier findings from a 1991–2009 study; this emphasizes the importance of preserving access to safe, effective, and economically viable oral candidiasis therapies in high-risk populations [2,12].

The introduction of efinaconazole in 2014 saw a sharp rise in its use, increasing by 9761% over the study period. This trend can be attributed to several factors, including its reported clinical efficacy in randomized trials, superior cure rates, enhanced nail penetration, and improved patient adherence when compared to older topical antifungals as a treatment for onychomycosis, a common fungal infection of the nail unit caused by dermatophytes, yeasts, and non-dermatophyte molds [13]. Its favorable safety profile and minimal systemic absorption may further contribute to its growing preference among prescribers. In this context, pharmacists play a crucial role as medicinal product specialists by ensuring the appropriate dispensing of antifungals, particularly newer agents such as efinaconazole, to optimize treatment outcomes and prevent misuse. 

Although this study focused on consumption trends and did not assess clinical outcomes directly, the increased use of efinaconazole may reflect heightened confidence in its therapeutic value. However, the utility of efinaconazole among Medicare patients has been limited by its high cost—averaging over USD 1000 per prescription—restricted insurance coverage, lower accessibility, and the availability of more affordable alternatives. Similarly, a study on Medicare patients supports these findings and highlights the need for strategies to improve affordability and insurance coverage, allowing broader access to essential treatments [14]. In contrast, older agents such as butoconazole and oxiconazole saw declining use. This trend not only reflected a preference for newer agents but also raised concerns about the future availability and clinical relevance of older therapies, especially in treating resistant cases [15].

The rise in butoconazole utilization in 2013 may be attributed to improvements in drug formulations, such as sustained-release, single-dose vaginal creams, enhancing convenience and compliance for vulvovaginal candidiasis (VVC) [16]. Additionally, a systematic review was conducted using published articles between 1990 and 2013, which showed the importance of accurate diagnostic techniques to differentiate VVC from other causes of vaginitis, reduce inappropriate treatments, and encourage targeted antifungal use [17]. Conversely, both oxiconazole and naftifine utilization saw a decline starting in 2012, driven by a shift toward more accessible and cost-effective alternatives like ketoconazole and nystatin. This was further compounded by the FDA approval of Luliconazole (Luzu^®^) in 2013, offering superior efficacy, shorter treatment durations, and improved safety [18]. Market dynamics, including the expiration of patents and the availability of generic alternatives, could have shifted utilization patterns [19]. Furthermore, changes in clinical guidelines and increased awareness of antifungal resistance may have led healthcare providers to favor other treatment options over naftifine and oxiconazole [20]. These trends align with broader reviews of antifungal advancements [21,22].

For invasive fungal infections (IFIs), fluconazole emerged as the most commonly prescribed agent, with 37.22 million prescriptions recorded during the study period. Its sustained dominance aligns with a previous study’s findings, which covered the period from 1991 to 2009 [2]. Its status as the most commonly used antifungal in the US is also supported by findings from a 2018 retrospective study on outpatient antifungal prescribing patterns [23]. Its established safety profile and proven effectiveness against common infections such as candidiasis and aspergillosis have sustained its popularity. Despite the challenges posed by fungal resistance, fluconazole continues to be widely used, as noted in earlier studies [2]. Additionally, its cost-effectiveness and availability in oral formulations make it more accessible, especially in outpatient and resource-constrained settings. Moreover, the rising number of immunocompromised patients has contributed to its increased use, as improved life expectancy has led to a greater need for antifungal treatments [24]. The notable reduction in the utilization of itraconazole in 2010, coupled with a corresponding increase in fluconazole’s prescriptions, may suggest a shift in Medicaid’s reimbursement strategy toward fluconazole. This shift could be attributed to fluconazole’s lower cost, which contributed to a reduction in the overall Medicaid reimbursement expenditures for IFI medications in that year. This trend is further supported by an earlier study that showed a consistent increase in the use of generic fluconazole after its introduction to the market. This growth remained stable in 2009 despite the introduction of newer antifungal agents, with approximately 1 million prescriptions issued. In contrast, the utilization of branded itraconazole steadily decreased after 2000 [2], a decline that may be attributed to the FDA’s 2001 black box warning regarding the risk of congestive heart failure associated with its use [25]. This warning likely impacted clinical practice and altered prescribing patterns for itraconazole. In the meantime, isavuconazole, first prescribed in 2015, showed a notable increase, rising from 102 prescriptions in its first year to 5732 by 2023. This growth demonstrates its ability to cure invasive aspergillosis and mucormycosis, comparable to voriconazole’s degree of efficacy but with fewer side effects [26]. For individuals taking several drugs, its simpler drug interaction profile enhances safety [27].

In 2011, our study observed a sudden spike in antifungal prescriptions, with an increase of 62% for superficial fungal infections and 57% for invasive fungal infections. This coincided with external data indicating a spike in the cases of coccidioidomycosis (Valley Fever) in 2011, particularly in the states of Arizona (66%) and California (31%) [28]. Therefore, these trends underscore the growing burden of fungal infections and the critical role of antifungal therapies.

### 3.2. Flucytosine

Flucytosine, a vital agent in the treatment of cryptococcal meningitis, faces significant challenges that limit its utilization and availability [29]. Among IFI medications, it has the lowest utilization rates within the Medicaid population, with only 1531 prescriptions during the study period. Additionally, it was the most expensive IFI medication, with its price substantially increasing over the initial years, peaking at USD 14,090 in 2016, followed by a decline, reaching USD 1903 in 2023. High production costs, complex manufacturing processes, and limited incentives for pharmaceutical companies have contributed to these barriers. Milan (now Viatris) is the only company with a WHO-prequalified flucytosine tablet product since March 2018. In 2020, Mylan relocated its production facility to India to reduce costs. However, an inspection at Legacy Pharmaceuticals Switzerland revealed a Good Manufacturing Practice (GMP) compliance failure related to product sterility. This led to a shutdown of Legacy Pharmaceuticals, creating a critical supply shortage of IV flucytosine. The resulting shortage severely impacted the supply of flucytosine in the United States, with tablets facing delivery delays of several weeks [30]. These disruptions may have contributed to the lack of utilization and reimbursement data for flucytosine in 2018 and 2020 in the Medicaid database. Moreover, economic barriers and regulatory challenges exacerbate these difficulties, restricting access to this life-saving drug [31]. Addressing these challenges requires coordinated global efforts, including increased investment in production, development of affordable generics, and stronger international partnerships to stabilize prices and ensure equitable access to this life-saving drug.

### 3.3. Market Dynamics and Economic Implications

Antifungal agents’ pricing and utilization trends reflect the market’s evolving nature and economic consequences. Newer therapies, such as efinaconazole, have maintained high prices, with a 147% price increase observed during the study period. Limited generic availability and clinical benefits have driven these elevated costs, notably impacting healthcare budgets. Fungal infections have been estimated to cost the US healthcare system USD 11.5 billion annually, with USD 7.5 billion in direct medical expenses [32]. On the other hand, older agents like clotrimazole and miconazole experienced price reductions due to increased generic competition. FDA reports confirm that generic drug entry substantially lowers prices, improving patient access [33]. Medicaid expenditures on SFI medications increased steadily, rising from USD 121.9 million in 2009 to USD 154.9 million in 2023. This growth is attributed to the recent rising prevalence of SFIs, which has placed a greater financial burden on healthcare systems, including Medicaid [14].

Medicaid’s highest expenditure on SFI medications for nystatin can be attributed to its extensive utilization among beneficiaries, which peaked at USD 91 million in 2013. The most contributing factor is the price increase; a Business Insider article reports that several drug manufacturers coordinated to raise the price of nystatin in 2014, with discussions about the increase beginning as early as mid-2013 [34]. This initial surge in spending was followed by a decline in Medicaid reimbursement for nystatin, driven by a combination of shifts in drug utilization patterns and systemic challenges in healthcare reimbursement. Medicaid outpatient prescription drug utilization peaked in FY 2017 but subsequently declined, driven by the increased use of generic alternatives and cost-containment measures, such as negotiated supplemental rebates and the promotion of cost-effective therapies [35]. Additionally, specific nystatin products, like pastilles (Nystan^®^), were discontinued commercially, while manufacturers such as Leading Pharma and Wockhardt faced shortages or ceased production entirely, limiting availability and reimbursement opportunities [36]. Market withdrawals by companies like Akorn and broader challenges, such as rising claim denials and complex payer policies, further exacerbated reimbursement barriers [37,38]. These combined factors highlight the challenges in ensuring consistent access to and coverage for older, low-cost medications like nystatin.

For IFI medications, posaconazole remained the second most expensive drug until 2020, when its price declined and isavuconazole’s price growth surpassed it. The decrease in posaconazole’s price can be attributed mainly to its patent expiration and FDA generic approval for oral formulation in 2019 [39,40]. A cost-effectiveness analysis (CUA) study published in 2025 conducting a cost–consequence analysis (CCA) comparing isavuconazole, posaconazole, and voriconazole for the treatment of invasive aspergillosis in China found that while isavuconazole had higher overall medical costs than posaconazole, it resulted in lower out-of-pocket expenses for patients, with no significant differences in clinical outcomes. Consequently, isavuconazole is likely the most economical choice among the three [41]. Other CUA studies conducted in Canada and Sweden using a decision tree analysis showed that isavuconazole is more cost effective than voriconazole in the treatment of invasive aspergillosis [42,43]. However, a pharmacoeconomic review report for isavuconazole published by the Canadian Agency for Drugs and Technology in Health demonstrated that isavuconazole is not cost effective compared with voriconazole, at a willingness to pay USD 50,000 per QALY unless the price of isavuconazole is reduced by at least 20% [44].

### 3.4. Clinical and Public Health Implications

Adopting new antifungal therapies such as efinaconazole underscores the importance of addressing specific patient needs through therapeutic innovation. Superior outcomes, such as higher cure rates and better compliance, demonstrate the value of these therapies in treating challenging infections like onychomycosis [45]. Meanwhile, the consistent reliance on nystatin highlights its role as an affordable and effective treatment, especially in cost-conscious healthcare systems [12].

Invasive fungal infections (IFIs) similarly highlight the need for effective antifungal agents. In this context, fluconazole’s cost effectiveness and broad-spectrum efficacy have made it a preferred treatment for various fungal infections, including candidemia. Its consistent use in both outpatient and inpatient settings across diverse populations reinforces its significance in antifungal therapy [46].

According to Miceli and Kauffman (2015), Maertens et al. (2016), and Marty et al. (2016), isavuconazole, a second-generation, broad-spectrum triazole antifungal, offers a favorable safety profile, predictable pharmacokinetics, and in vitro activity against molds, yeasts, and dimorphic fungi. It is generally well tolerated and associated with fewer drug–drug interactions than older triazoles and has demonstrated non-inferiority to voriconazole in the treatment of invasive aspergillosis, with the additional benefits of once-daily dosing and availability in both intravenous and oral formulations [26,47,48].

While we acknowledge that the absence of diagnosis information in prescription claims limits our ability to determine the exact clinical indication for antifungal use, the observed co-prescription trends with broad-spectrum antibiotics may indirectly reflect prophylactic practices in high-risk populations.

### 3.5. Impact of the COVID-19 Pandemic

The COVID-19 pandemic had a global influence on antifungal prescriptions, with our data showing a decline in 2020 and a spike in 2022 due to an increase in secondary fungal infections [49]. This trend was observed globally, with the UK seeing a 19.4% decline in antifungal usage from 2019 to 2021, suggesting changes in healthcare priorities, while the US saw a 60% increase in Candida auris infections, underscoring the challenges of antifungal resistance [11,50]. Similarly, a retrospective analysis revealed a notable increase in Candida auris incidence throughout the epidemic. From 2.6 per 10,000 admissions in 2019 to 7.8 per 10,000 in 2022, incidence rates surged [51]. Another study indicated that recent COVID-19 infection was associated with 23.4% of mucormycosis cases, primarily during 2021, while only a small number were observed during 2022 [52], indicating that the risk of fungal infection may have been raised by the severity of the sickness and the treatment methods used at the time. This highlights the importance of strong healthcare systems and resilient pharmaceutical supply chains to ensure continuity of care during global crises.

### 3.6. Policy Recommendations

To address rising costs and ensure equitable access to antifungal drugs, this study highlights the need for comprehensive antifungal stewardship programs. These measures should focus on:

- Promoting generic substitution: Encourage using affordable generics to reduce spending without compromising treatment quality [33].
- Conducting cost-effectiveness analyses: Regularly evaluate newer therapies to ensure they provide added clinical value relative to their costs [11].
- Monitoring resistance: Strengthen diagnostic tools and surveillance systems to monitor antifungal resistance trends, particularly in low- and middle-income populations [45].
- Enhancing education and awareness: Initiate education programs for healthcare providers on optimal prescribing practices to combat resistance and improve treatment outcomes [11].

### 3.7. Limitations

This study relied on prescription and reimbursement data, which lacked detailed clinical outcomes and off-label use information. The absence of patient-specific data, such as demographics, geographic location, and prescription duration, restricted the ability to analyze prescribing patterns across subpopulations and treatment adherence. Additionally, the lack of granular resistance data and reliance on secondary sources may not fully reflect current antifungal resistance challenges. One of the main limitations was the use of “reimbursement per prescription” as a proxy for drug price, which may not accurately reflect actual pricing due to variations in dosage forms, treatment durations, and packaging sizes. This approach could introduce bias or obscure significant differences in medication costs. Furthermore, external factors such as healthcare disruptions (e.g., the COVID-19 pandemic) and market dynamics, including pharmaceutical marketing or policy changes, could have impacted prescribing trends. Future research should integrate comprehensive patient-level data, resistance surveillance, and real-world outcomes to enhance our understanding of antifungal utilization and its broader implications for policy and stewardship [46]. 

## 4. Materials and Methods

### 4.1. Study Design

A retrospective drug utilization analysis of antifungal medications was conducted over the period from 2009 to 2023 using data obtained from a national Medicaid pharmacy claims database provided by the Centers for Medicare & Medicaid Services (CMS). Medicaid, one of the largest healthcare payers in the United States, provides coverage to low-income individuals [53]. Each record in the dataset included an 11-digit National Drug Code (NDC), covering both the brand and generic names of the drug, as well as information on the quarter and year of Medicaid expenditure, the number of outpatient prescription claims, the quantity of units (dose units), and the total pharmacy reimbursement. Incomplete records were excluded from the analysis.

A comprehensive database search was conducted to identify all currently approved and available antifungal medications using their brand names and National Drug Codes (NDCs) provided in File S1, Supplementary. Relevant NDCs were identified using the brand and generic names of antifungal drugs. These codes were then used to extract CMS Medicaid outpatient claims data. The antifungal medications were classified based on therapeutic use into two classes: superficial fungal infections’ (SFIs’) medications, including nystatin, clotrimazole, ketoconazole, terconazole, terbinafine, griseofulvin, ciclopirox, miconazole, econazole, oxiconazole, naftifine, efinaconazole, butoconazole, butenafine, natamycin, and tioconazole, and invasive fungal infections’ (IFIs’) medications, including fluconazole, itraconazole, voriconazole, micafungin, posaconazole, amphotericin B, caspofungin, isavuconazole, anidulafungin, and flucytosine.

Although they are classified as medications for invasive fungal infections, most of these medications are also used to treat some types of superficial fungal infections, such as oropharyngeal and esophageal candidiasis [54,55].

### 4.2. Data Analysis

The study focused on the following analytical aspects:

1. Utilization: Annual utilization was obtained by summing the number of prescriptions for each drug. This category provides a valuable understanding of prescribing patterns and utilization trends.
2. Reimbursement: Total Medicaid expenditures on each antifungal medication were analyzed, providing insights into the financial impact of these drugs on the healthcare system. Annual reimbursement values were calculated, with all figures expressed in US dollars.
3. Price proxy: The reimbursement per antifungal medication was estimated by dividing the total reimbursement by the number of prescriptions for each drug. This measure is an approximate indicator of the medication’s price and helps assess the pricing and affordability.
4. Market Share: Market share for utilization and reimbursement was determined by dividing each drug’s prescription or spending by the total utilization or spending for all antifungal medications within the corresponding therapeutic class in a given year. This analysis provided a clearer understanding of each medication’s market position and financial effect within the Medicaid program.

All statistical analyses were conducted using the SAS software package for Windows (version 9.4) and Microsoft Excel Pro Plus 2021 (version 2108). A trend analysis was performed using Microsoft Excel by plotting the data for each parameter over time using a line chart with markers. The lines for each parameter either rose, fell, or remained constant, allowing for a straightforward interpretation of the trends.

To detect changes in the direction of these trends, Joinpoint regression analyses were employed using Joinpoint Trend Analysis Software (version 5.3.0). The empirical quantile method was used to address non-normal distributions and outliers, allowing for estimating regression parameters based on the data’s specific distribution. Standard error was incorporated into the models, with a significance level of 0.05 established for accuracy. The software provided both the annual percent change (APC) and the average annual percent change (AAPC) at each joinpoint, offering a detailed assessment of the rate of change in the variables over time.

## 5. Conclusions

This study underscores a significant rise in Medicaid utilization and spending on antifungal medications from 2009 to 2023, driven by the increasing demand for effective fungal infection management. While nystatin and fluconazole remain essential due to their affordability and effectiveness, newer antifungal agents, such as efinaconazole and isavuconazole, offer promising clinical benefits but are limited by high costs.

## Figures and Tables

**Table 1:** SFI medications’ utilization (number of prescriptions) from 2009 to 2023.

Year	Tioconazole	Miconazole	Clotrimazole	Butenafine	Ketoconazole	Oxiconazole	Terconazole	Butoconazole	Efinaconazole	Econazole	Naftifine	Terbinafine	Ciclopirox	Griseofulvin	Nystatin	Natamycin	Total/Year
2009	50	158,924	910,654	5539	449,860	77,152	258,927	16,769	-	96,638	44,928	131,686	104,715	207,792	1,484,494	254	3,948,382
2010	108	136,958	984,560	2914	544,555	73,702	271,535	350	-	83,538	37,108	143,314	76,743	224,963	1,660,941	204	4,241,493
2011	165	236,013	1,608,802	4017	946,367	74,730	438,626	105	-	145,565	49,096	243,060	169,955	358,950	2,608,465	280	6,884,196
2012	146	209,432	1,629,118	1375	1,093,399	19,082	443,009	10	-	121,854	15,177	246,120	135,655	359,785	2,716,194	352	6,990,708
2013	106	187,326	1,607,009	1066	1,122,323	6880	410,799	2499	-	124,008	8400	249,758	133,730	306,544	2,631,793	294	6,792,535
2014	103	179,729	1,543,355	941	1,182,534	5468	385,284	1473	208	105,674	5706	309,799	120,318	290,620	2,547,087	436	6,678,735
2015	11	209,919	1,879,073	713	1,473,189	4942	394,004	1703	4575	145,546	3084	433,492	186,995	268,965	2,657,319	622	7,664,152
2016	53	202,883	2,008,253	591	1,642,419	4151	419,582	5129	5791	84,380	2605	467,128	234,081	237,383	2,652,297	600	7,967,326
2017	39	193,417	1,953,023	541	1,826,200	3357	371,088	6655	5745	66,259	2146	452,894	233,354	210,005	2,532,069	706	7,857,498
2018	52	170,310	1,912,268	1161	1,770,567	2615	354,647	5081	4736	62,040	3904	448,995	223,960	194,009	2,403,160	632	7,558,137
2019	11	157,593	1,776,249	1378	1,756,410	3183	328,168	4789	4758	43,040	2972	446,978	213,775	159,964	2,186,090	700	7,086,058
2020	27	157,379	1,368,141	1641	769,209	2910	273,640	4082	5356	39,004	3719	315,756	232,813	124,808	1,686,287	754	4,985,526
2021	25	147,213	1,402,516	1588	770,906	2285	307,679	2619	5894	37,334	4710	315,237	269,168	109,739	1,731,361	657	5,108,931
2022	13	199,785	2,462,040	2842	3,136,586	6030	443,350	2977	15,533	51,000	5630	749,177	454,483	144,934	3,205,603	361	10,880,344
2023	23	148,959	1,629,668	2140	1,063,645	9781	298,705	1749	20,511	30,597	4250	435,297	373,097	91,914	2,049,802	272	6,160,410
Total/drug	932	2,695,840	24,674,729	28,447	19,548,169	296,268	5,399,043	55,990	73,107	1,236,477	193,435	5,388,691	3,162,842	3,290,375	34,752,962	7124	–

**Table 2:** IFI medications’ utilization (number of prescriptions) from 2009 to 2023.

Year	Amphotericin B	Fluconazole	Itraconazole	Voriconazole	Posaconazole	Isavuconazole	Caspofungin	Micafungin	Anidulafungin	Total/Year
2009	2568	1,091,736	215,261	7876	1386	-	3892	2302	356	1,325,377
2010	3417	1,385,916	27,047	8513	1528	-	4150	3094	287	1,433,952
2011	5154	2,188,189	29,792	12,837	2128	-	3924	4985	160	2,247,169
2012	5796	2,337,061	26,151	13,141	2227	-	3098	5981	106	2,393,561
2013	6092	2,323,768	22,705	13,460	2221	-	2284	6245	81	2,376,856
2014	5598	2,587,887	24,371	13,642	3378	-	3246	6462	202	2,644,786
2015	6795	3,115,656	26,185	12,855	4784	102	2606	6160	210	3,175,353
2016	5659	3,284,826	26,925	13,235	5813	886	2974	7847	310	3,348,475
2017	5813	3,347,707	25,329	13,138	6653	1721	2293	8141	431	3,411,226
2018	4609	3,314,100	21,517	13,390	6878	2569	1721	8350	337	3,373,471
2019	4459	3,239,675	19,934	13,231	7296	2908	1547	8513	629	3,298,192
2020	5747	1,054,699	19,205	13,754	8257	3271	1456	9602	645	1,116,636
2021	5222	1,156,917	19,958	13,294	9260	3870	1452	10,721	993	1,221,687
2022	6064	5,551,425	31,648	15,820	16,478	6524	1461	13,702	1433	5,644,555
2023	3693	1,248,183	24,933	11,497	8479	5732	667	10,905	508	1,314,597
Total/drug	76,686	37,227,745	560,961	189,683	86,766	27,583	36,771	113,010	6688	-

**Table 3:** CMS spending (reimbursement) for SFI medications from 2009 to 2023 in US dollars.

Year	Tioconazole	Miconazole	Clotrimazole	Butenafine	Ketoconazole	Oxiconazole	Terconazole	Butoconazole	Efinaconazole	Econazole	Naftifine	Terbinafine	Ciclopirox	Griseofulvin	Nystatin	Natamycin	Total/Year
2009	706	13,870,666	22,636,021	546,170	11,861,647	7,425,590	8,621,016	1,143,809	–	3,283,806	5,508,397	2,441,733	6,465,920	20,481,863	17,525,944	47,874	121,861,160
2010	1584	8,220,521	20,544,577	313,523	13,152,716	8,330,630	8,173,764	23,471	–	2,462,326	5,450,399	2,276,685	4,764,734	20,244,808	17,942,333	38,918	111,940,989
2011	2651	9,872,862	38,866,136	491,782	20,366,980	10,097,443	11,584,083	6821	–	4,786,719	11,248,769	3,923,861	8,413,900	30,699,449	37,149,199	57,247	187,567,903
2012	2337	4,583,901	45,346,978	203,076	22,114,898	3,038,308	10,326,382	651	–	3,581,737	4,440,692	3,410,953	6,519,472	34,374,613	73,217,674	81,671	211,243,343
2013	1288	3,213,956	42,632,166	141,714	20,625,971	1,444,927	11,561,296	206,157	–	3,395,118	2,513,250	3,147,246	6,290,660	27,741,398	91,047,522	74,738	214,037,407
2014	1538	2,810,386	38,061,934	131,291	23,763,892	1,549,292	13,491,288	127,062	92,298	5,722,933	1,924,730	3,599,755	5,379,857	25,119,105	79,595,652	117,025	201,488,038
2015	139	3,094,047	42,160,058	117,939	41,935,013	2,007,740	13,170,951	155,059	2,626,911	29,891,405	1,138,999	4,321,840	7,557,709	24,337,737	75,224,990	180,412	247,920,949
2016	786	2,834,676	45,018,523	104,294	58,624,717	1,695,775	13,302,572	487,974	3,847,545	14,397,985	912,495	4,940,325	9,908,787	21,085,541	68,991,764	178,699	246,332,457
2017	613	2,482,612	39,005,101	95,627	58,617,844	1,299,280	11,393,125	661,340	4,375,619	10,090,685	823,588	4,326,978	9,301,681	21,011,328	57,248,808	220,040	220,954,270
2018	689	2,209,952	31,571,946	47,350	43,989,450	1,028,460	9,565,634	523,894	3,309,337	8,020,234	1,586,240	4,162,894	5,753,897	17,925,710	43,714,962	209,694	173,620,342
2019	144	2,367,549	29,220,861	37,484	42,742,536	1,898,683	10,154,584	517,193	3,629,364	2,872,375	996,578	4,206,659	6,334,898	15,718,460	36,503,323	230,976	157,431,664
2020	338	2,305,130	21,201,066	22,095	24,678,441	1,504,616	8,832,370	439,658	4,188,889	1,686,786	1,159,699	3,292,517	5,695,879	13,324,753	26,287,231	246,702	114,866,169
2021	251	1,894,727	22,294,470	18,618	22,082,137	923,370	9,449,224	291,736	4,963,970	1,663,513	1,517,766	3,431,970	6,525,394	12,569,284	26,560,573	257,629	114,444,630
2022	145	3,993,528	39,787,485	108,059	68,137,151	4,223,385	13,541,882	348,152	14,666,093	2,220,708	1,488,841	8,747,323	11,729,899	17,758,057	47,782,586	175,321	234,708,615
2023	434	2,301,197	26,304,217	35,561	26,788,524	5,347,041	9,539,909	228,744	22,445,920	1,353,506	1,363,055	5,411,197	9,058,190	14,082,867	30,569,072	129,046	154,958,479
Total/drug	13,645	66,055,709	504,651,537	2,414,583	499,481,915	51,814,539	162,708,079	5,161,721	64,145,945	95,429,837	42,073,496	61,641,937	109,700,878	316,474,972	729,361,631	2,245,991	–

**Table 4:** CMS spending (reimbursement) for IFI medications from 2009 to 2023 in US dollars.

Year	Amphotericin B	Fluconazole	Itraconazole	Voriconazole	Posaconazole	Isavuconazole	Caspofungin	Micafungin	Anidulafungin	Total/Year
2009	4,526,613	14,602,241	75,780,168	14,084,316	3,368,296	–	5,387,264	1,713,485	179,767	119,642,150
2010	4,001,320	13,960,769	10,654,491	15,438,043	3,709,108	–	6,263,865	2,166,557	173,769	56,367,922
2011	6,092,378	20,054,742	13,097,376	22,692,903	5,814,780	–	5,988,038	3,806,293	133,914	77,680,425
2012	8,742,803	19,358,500	12,590,339	22,349,141	6,570,437	–	5,043,508	4,297,008	106,078	79,057,814
2013	10,243,780	20,894,652	11,039,246	22,362,293	8,050,177	–	3,520,937	4,468,666	50,648	80,630,399
2014	8,383,540	29,954,095	11,623,607	20,811,932	13,224,262	–	4,901,340	4,780,272	116,602	93,795,651
2015	8,753,187	34,014,871	12,631,776	18,117,864	21,139,723	347,268	3,360,416	4,592,674	132,970	103,090,749
2016	9,089,800	31,856,838	12,761,871	15,005,203	27,471,714	3,129,377	3,583,272	5,847,899	204,425	108,950,398
2017	9,374,131	31,348,521	11,688,844	12,026,984	33,201,863	7,083,453	3,000,105	6,160,942	295,501	114,180,345
2018	11,232,001	28,987,241	9,923,398	11,976,817	33,207,376	10,698,479	1,814,307	5,715,383	165,891	113,720,894
2019	7,996,032	28,391,204	8,655,503	12,176,887	37,727,227	13,433,130	1,086,102	5,663,606	239,917	115,369,609
2020	11,511,857	15,717,919	8,105,308	13,264,587	33,161,041	15,236,511	1,092,367	5,668,718	241,671	103,999,978
2021	10,131,992	16,706,564	7,134,834	9,281,145	29,681,631	18,740,791	659,094	5,400,513	539,835	98,276,401
2022	12,404,587	46,510,342	7,002,900	7,372,841	40,834,356	34,563,092	653,816	6,880,876	531,609	156,754,420
2023	6,900,370	15,950,015	4,314,678	4,474,403	13,285,476	30,589,727	219,347	4,805,252	169,795	80,709,064
Total/drug	129,384,390	368,308,515	217,004,341	221,435,360	310,447,470	133,821,828	46,573,778	71,968,144	3,282,392	–

**Table 5:** Prices (reimbursement per prescription) for SFI medications from 2009 to 2023 in US dollars.

Year	Tioconazole	Miconazole	Clotrimazole	Butenafine	Ketoconazole	Oxiconazole	Terconazole	Butoconazole	Efinaconazole	Econazole	Naftifine	Terbinafine	Ciclopirox	Griseofulvin	Nystatin	Natamycin	
2009	14.13	87.28	24.86	98.60	26.37	96.25	33.30	68.21	-	33.98	122.60	18.54	61.75	98.57	11.81	188.48	
2010	14.67	60.02	20.87	107.59	24.15	113.03	30.10	67.06	-	29.48	146.88	15.89	62.09	89.99	10.80	190.77	
2011	16.07	41.83	24.16	122.43	21.52	135.12	26.41	64.96	-	32.88	229.12	16.14	49.51	85.53	14.24	204.46	
2012	16.01	21.89	27.84	147.69	20.23	159.22	23.31	65.07	-	29.39	292.59	13.86	48.06	95.54	26.96	232.02	
2013	12.15	17.16	26.53	132.94	18.38	210.02	28.14	82.50	-	27.38	299.20	12.60	47.04	90.50	34.60	254.21	
2014	14.94	15.64	24.66	139.52	20.10	283.34	35.02	86.26	443.74	54.16	337.32	11.62	44.71	86.43	31.25	268.41	
2015	12.68	14.74	22.44	165.41	28.47	406.26	33.43	91.05	574.19	205.37	369.33	9.97	40.42	90.49	28.31	290.05	
2016	14.82	13.97	22.42	176.47	35.69	408.52	31.70	95.14	664.40	170.63	350.29	10.58	42.33	88.82	26.01	297.83	
2017	15.72	12.84	19.97	176.76	32.10	387.04	30.70	99.37	761.64	152.29	383.78	9.55	39.86	100.05	22.61	311.67	
2018	13.26	12.98	16.51	40.78	24.84	393.29	26.97	103.11	698.76	129.28	406.31	9.27	25.69	92.40	18.19	331.79	
2019	13.06	15.02	16.45	27.20	24.34	596.51	30.94	108.00	762.79	66.74	335.32	9.41	29.63	98.26	16.70	329.97	
2020	12.54	14.65	15.50	13.46	32.08	517.05	32.28	107.71	782.09	43.25	311.83	10.43	24.47	106.76	15.59	327.19	
2021	10.05	12.87	15.90	11.72	28.64	404.10	30.71	111.39	842.21	44.56	322.24	10.89	24.24	114.54	15.34	392.13	
2022	11.13	19.99	16.16	38.02	21.72	700.40	30.54	116.95	944.19	43.54	264.45	11.68	25.81	122.53	14.91	485.65	
2023	18.87	15.45	16.14	16.62	25.19	546.68	31.94	130.79	1094.34	44.24	320.72	12.43	24.28	153.22	14.91	474.44	

**Table 6:** Prices (reimbursement per prescription) for IFI medications from 2009 to 2023 in US dollars.

Year	Amphotericin B	Fluconazole	Itraconazole	Voriconazole	Posaconazole	Isavuconazole	Caspofungin	Micafungin	Anidulafungin
2009	1762.70	13.38	352.04	1788.26	2430.23	-	1384.19	744.35	504.96
2010	1171.00	10.07	393.93	1813.47	2427.43	-	1509.36	700.24	605.47
2011	1182.07	9.16	439.63	1767.77	2732.51	-	1526.00	763.55	836.96
2012	1508.42	8.28	481.45	1700.72	2950.35	-	1627.99	718.44	1000.74
2013	1681.51	8.99	486.20	1661.39	3624.57	-	1541.57	715.56	625.28
2014	1497.60	11.57	476.94	1525.58	3914.82	-	1509.96	739.75	577.24
2015	1288.18	10.92	482.41	1409.40	4418.84	3404.59	1289.49	745.56	633.19
2016	1606.26	9.70	473.98	1133.75	4725.91	3532.03	1204.87	745.24	659.44
2017	1612.62	9.36	461.48	915.43	4990.51	4115.89	1308.38	756.78	685.62
2018	2436.97	8.75	461.19	894.46	4828.06	4164.45	1054.22	684.48	492.26
2019	1793.23	8.76	434.21	920.33	5170.95	4619.37	702.07	665.29	381.43
2020	2003.11	14.90	422.04	964.42	4016.11	4658.06	750.25	590.37	374.68
2021	1940.25	14.44	357.49	698.15	3205.36	4842.58	453.92	503.73	543.64
2022	2045.61	8.38	221.27	466.05	2478.11	5297.84	447.51	502.18	370.98
2023	1868.50	12.78	173.05	389.18	1566.87	5336.66	328.86	440.65	334.24

## Data Availability

CMS data are publicly available from the Centers for Medicare & Medicaid Services at https://www.medicaid.gov/medicaid/prescription-drugs/state-drug-utilization-data/index.html, accessed on 1 October 2024.

## Supplementary Materials

The following supporting information can be downloaded at https://www.mdpi.com/article/10.3390/antibiotics14050518/s1: Figure S1: Number of prescriptions for market share of SFIs’ medications from 2009–2023. Figure S2: Reimbursement market share for SFIs’ medications from 2009 to 2023. Figure S3: Reimbursement market share for IFIs’ medications from 2009 to 2023. Figure S4: Joinpoint regression for SFIs’ medications’ utilization. Figure S5: Joinpoint regression for SFIs’ medications’ spending. Figure S6: Joinpoint regression for SFIs’ medications’ price. Figure S7: Joinpoint regression for IFIs’ medications’ utilization. Figure S8: Joinpoint regression for IFIs’ medications’ spending. Figure S9: Joinpoint regression for IFIs’ medications’ prices. File S1: Antifungal NDC code nodups.
