# Supplementary material for: Antifungal Agents’ Trends of Utilization, Spending, and Prices in the US Medicaid Programs: 2009–2023
Source: Antibiotics (Basel). 2025 May 16;14(5):518. doi: 10.3390/antibiotics14050518 (PMC12108345; doi:10.3390/antibiotics14050518)
Supplement: Supplementary file 1 [file antibiotics-14-00518-s001.zip › Supplementary Materials.pdf]

Supplementary Materials

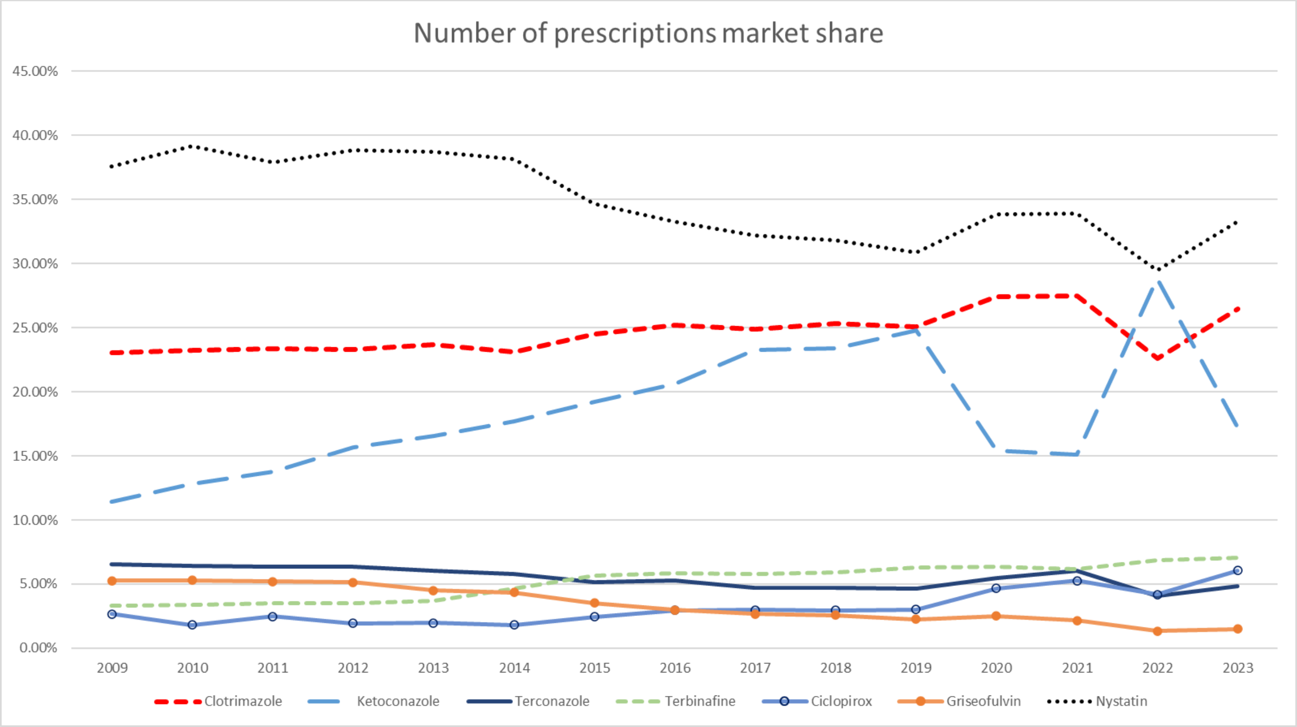

Figure S1: Number of prescriptions market share of SFIs' medications from 2009-2023.

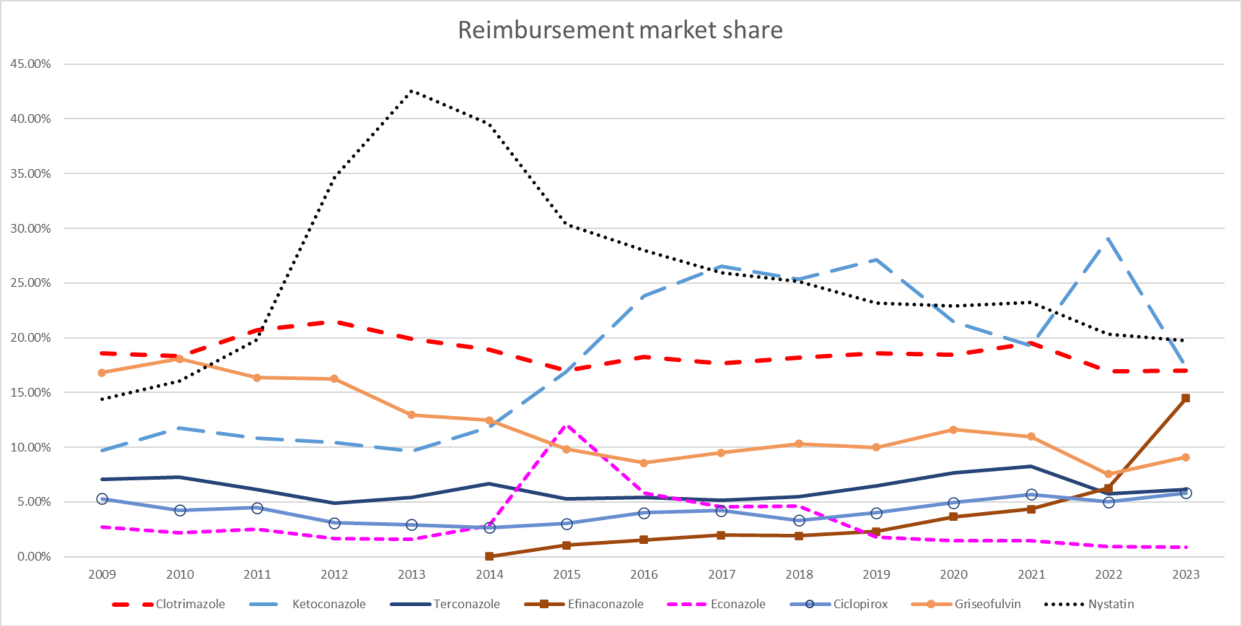

Figure S2 Reimbursement market share for SFIs' medications from 2009 to 2023.

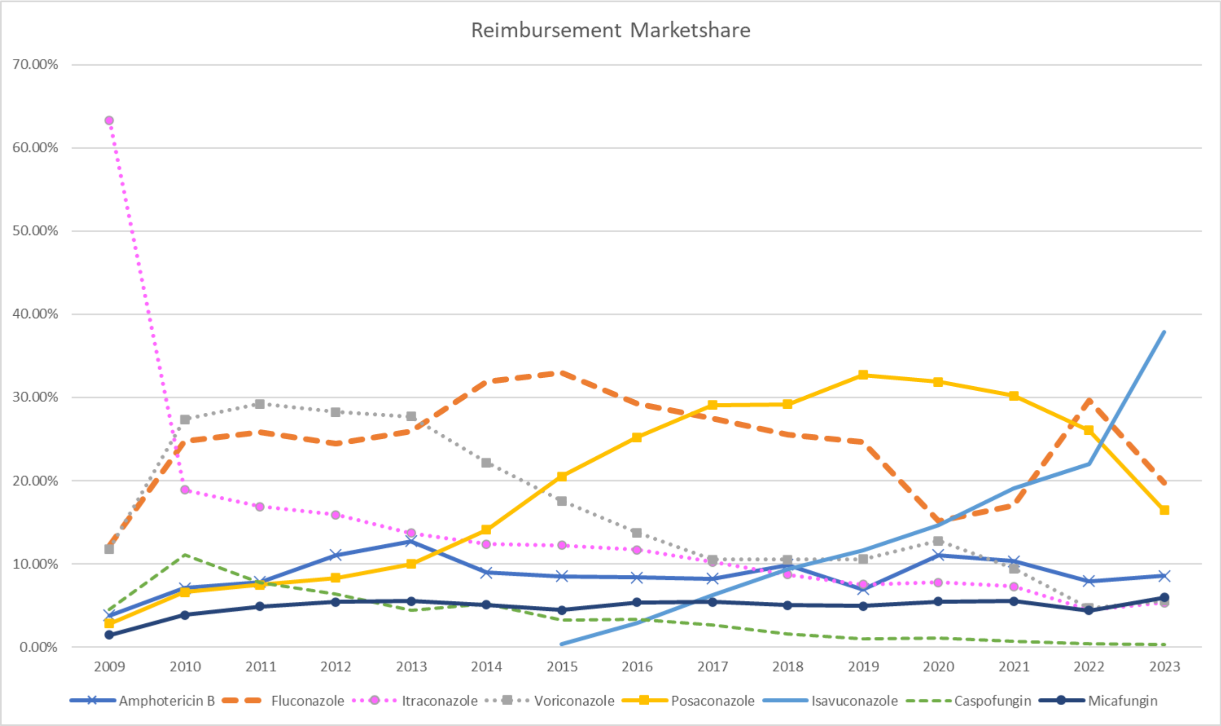

Figure S3: Reimbursement market share for IFIs medications from 2009 to 2023.

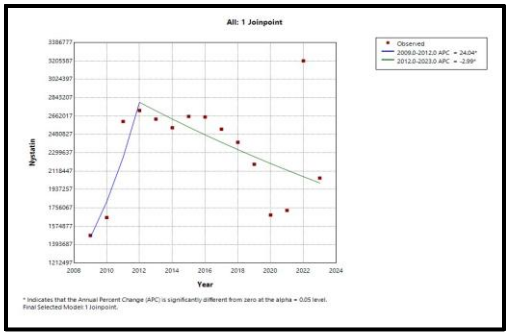
All: 1 Joinpoint
Nystatin
3386777
3205587
3024397
2843207
2662017
2480827
2299637
2118447
1937257
1756067
1574877
1393687
1212497
2008
2010
2012
2014
2016
2018
2020
2022
2024
Year
Observed
2009.0-2012.0 APC = 24.04*
2012.0-2023.0 APC = -2.99*
* Indicates that the Annual Percent Change (APC) is significantly different from zero at the alpha = 0.05 level.
Final Selected Model: 1 Joinpoint.

A)

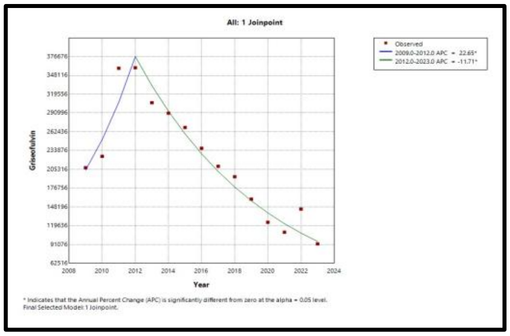
All: 1 Joinpoint
Griseofulvin
376676
348116
319556
290996
262436
233876
205316
176756
148196
119636
91076
62516
2008
2010
2012
2014
2016
2018
2020
2022
2024
Year
Observed
2009.0-2012.0 APC = 22.65*
2012.0-2023.0 APC = -11.71*
* Indicates that the Annual Percent Change (APC) is significantly different from zero at the alpha = 0.05 level.
Final Selected Model: 1 Joinpoint.

B)

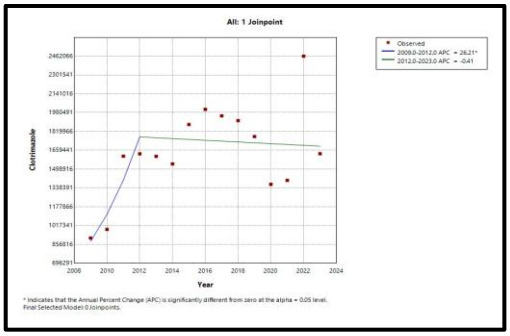
All: 1 Joinpoint
Clotrimazole
2462066
2301541
2141016
1980491
1819966
1659441
1498916
1338391
1177866
1017341
856816
696291
2008
2010
2012
2014
2016
2018
2020
2022
2024
Year
Observed
2009.0-2012.0 APC = 26.21*
2012.0-2023.0 APC = -0.41
* Indicates that the Annual Percent Change (APC) is significantly different from zero at the alpha = 0.05 level.
Final Selected Model: 0 Joinpoints.

C)

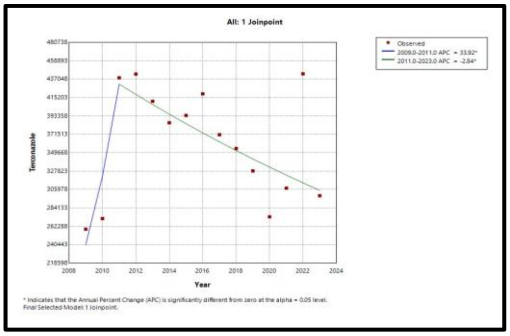
All: 1 Joinpoint
Terconazole
480738
458893
437048
415203
393358
371513
349668
327823
305978
284133
262288
240443
218598
2008
2010
2012
2014
2016
2018
2020
2022
2024
Year
Observed
2009.0-2011.0 APC = 33.92*
2011.0-2023.0 APC = -2.84*
* Indicates that the Annual Percent Change (APC) is significantly different from zero at the alpha = 0.05 level.
Final Selected Model: 1 Joinpoint.

D)

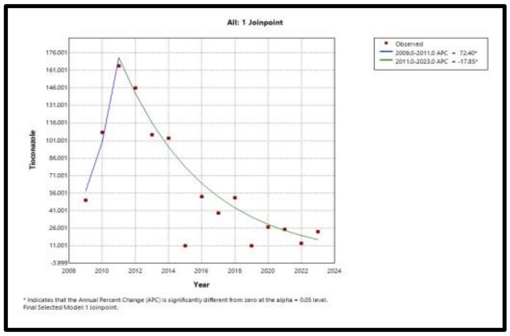
All: 1 Joinpoint
Tioconazole
176.001
161.001
146.001
131.001
116.001
101.001
86.001
71.001
56.001
41.001
26.001
11.001
-3.999
2008
2010
2012
2014
2016
2018
2020
2022
2024
Year
Observed
2009.0-2011.0 APC = 72.40*
2011.0-2023.0 APC = -17.85*
* Indicates that the Annual Percent Change (APC) is significantly different from zero at the alpha = 0.05 level.
Final Selected Model: 1 Joinpoint.

E)

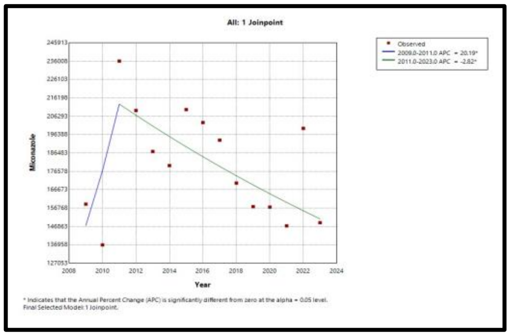
All: 1 Joinpoint
Miconazole
245913
236008
226103
216198
206293
196388
186483
176578
166673
156768
146863
136958
127053
2008
2010
2012
2014
2016
2018
2020
2022
2024
Year
Observed
2009.0-2011.0 APC = 20.19*
2011.0-2023.0 APC = -2.82*
* Indicates that the Annual Percent Change (APC) is significantly different from zero at the alpha = 0.05 level.
Final Selected Model: 1 Joinpoint.

F)

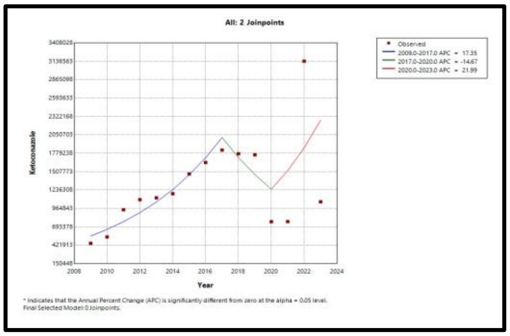
All: 2 Joinpoints
Ketoconazole
3408028
3136563
2865098
2593633
2322168
2050703
1779238
1507773
1236308
964843
693378
421913
150448
2008
2010
2012
2014
2016
2018
2020
2022
2024
Year
Observed
2009.0-2017.0 APC = 17.35
2017.0-2020.0 APC = -14.67
2020.0-2023.0 APC = 21.99
* Indicates that the Annual Percent Change (APC) is significantly different from zero at the alpha = 0.05 level.
Final Selected Model: 0 Joinpoints.

G)

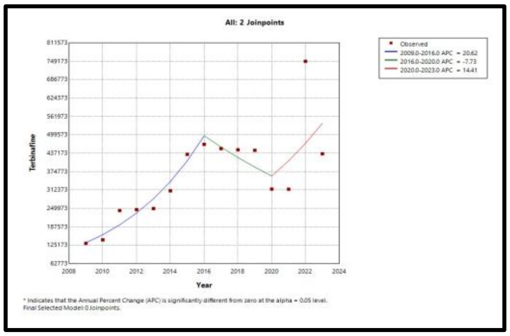
All: 2 Joinpoints
Terbinafine
811573
749173
686773
624373
561973
499573
437173
374773
312373
249973
187573
125173
62773
2008
2010
2012
2014
2016
2018
2020
2022
2024
Year
Observed
2009.0-2016.0 APC = 20.62
2016.0-2020.0 APC = -7.73
2020.0-2023.0 APC = 14.41
* Indicates that the Annual Percent Change (APC) is significantly different from zero at the alpha = 0.05 level.
Final Selected Model: 0 Joinpoints.

H)

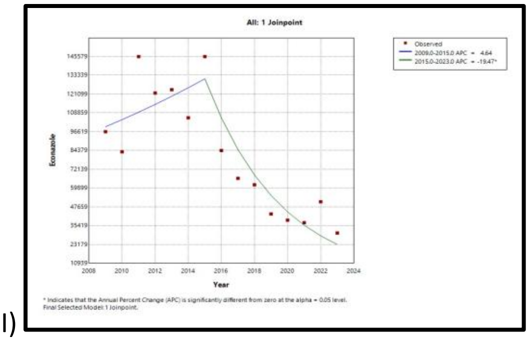

I)

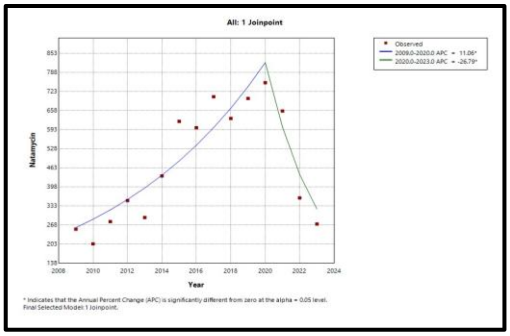

J)

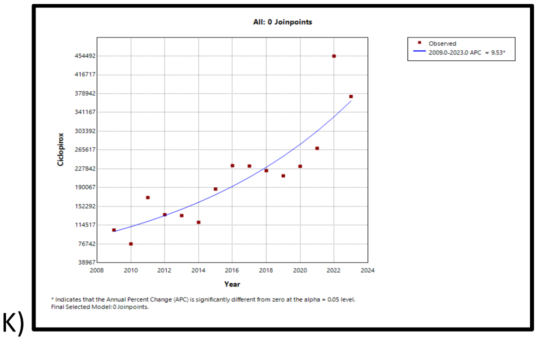

K)

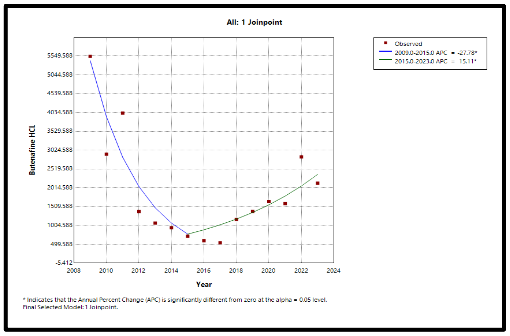

L)

Figure S4. Joinpoint regression for SFIs' medications' utilization. (A): Nystatin, (B): Griseofulvin, (C): Clotrimazole, (D): Terconazole, (E): Tioconazole, (F): Miconazole, (G): Ketoconazole, (H): Terbinafine, (I): Econazole, (J): Natamycin, (K): Ciclopirox, (L): Butenafine.

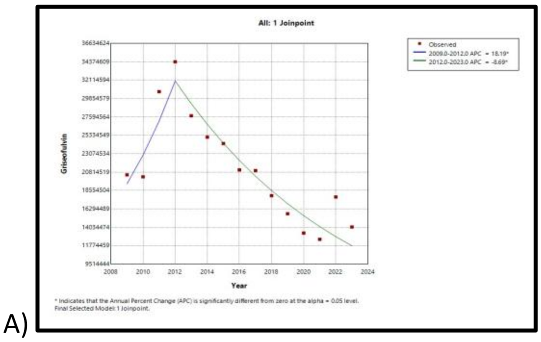

A)

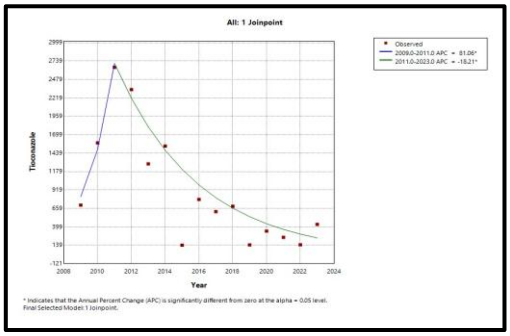

B)

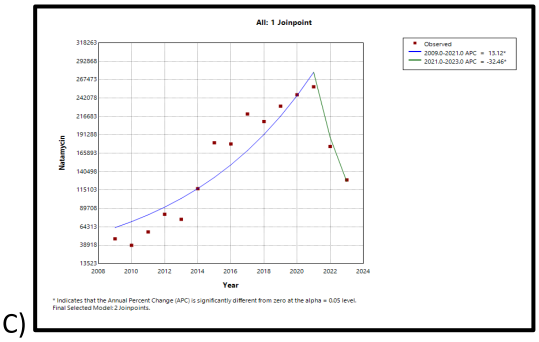

C)

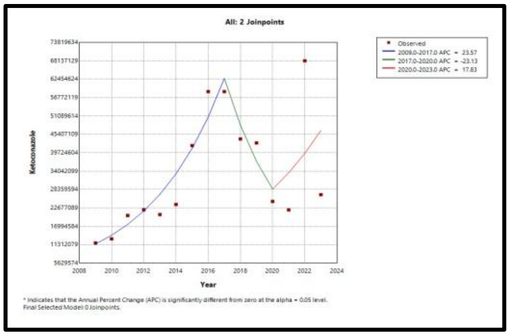

D)

E) 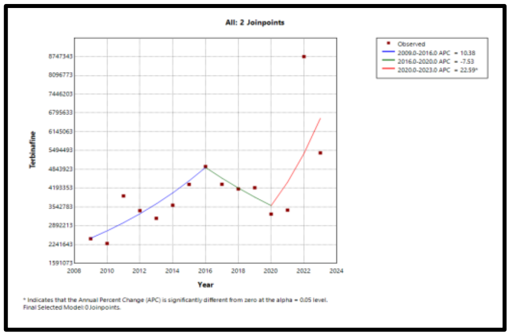
All: 2 Joinpoints
Terbinafine
8747343
8096773
7446203
6795633
6145063
5494493
4843923
4193353
3542783
2892213
2241643
1591073
2008 2010 2012 2014 2016 2018 2020 2022 2024
Year
Observed
2009.0-2016.0 APC = 10.38
2016.0-2020.0 APC = -7.53
2020.0-2023.0 APC = 22.59*
* Indicates that the Annual Percent Change (APC) is significantly different from zero at the alpha = 0.05 level.
Final Selected Model: 0 Joinpoints.

F) 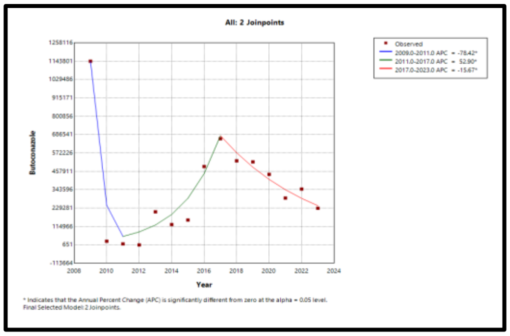
All: 2 Joinpoints
Butoconazole
1258116
1143801
1029486
915171
800856
686541
572226
457911
343596
229281
114966
651
-113664
2008 2010 2012 2014 2016 2018 2020 2022 2024
Year
Observed
2009.0-2011.0 APC = -78.42*
2011.0-2017.0 APC = 52.90*
2017.0-2023.0 APC = -15.67*
* Indicates that the Annual Percent Change (APC) is significantly different from zero at the alpha = 0.05 level.
Final Selected Model: 2 Joinpoints.

G) 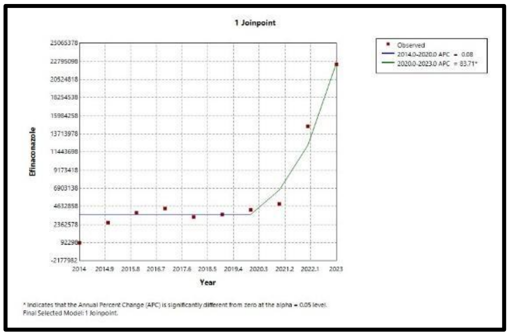
1 Joinpoint
Efinaconazole
25065378
22795098
20524818
18254538
15984258
13713978
11443698
9173418
6903138
4632858
2362578
92298
-2177982
2014 2014.9 2015.8 2016.7 2017.6 2018.5 2019.4 2020.3 2021.2 2022.1 2023
Year
Observed
2014.0-2020.0 APC = 0.08
2020.0-2023.0 APC = 83.71*
* Indicates that the Annual Percent Change (APC) is significantly different from zero at the alpha = 0.05 level.
Final Selected Model: 1 Joinpoint.

H) 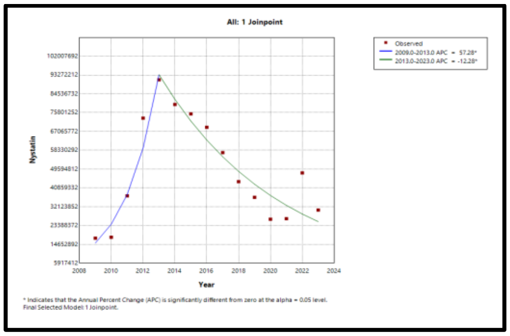
All: 1 Joinpoint
Nystatin
102007692
93272212
84536732
75801252
67065772
58330292
49594812
40859332
32123852
23388372
14652892
5917412
2008 2010 2012 2014 2016 2018 2020 2022 2024
Year
Observed
2009.0-2013.0 APC = 57.28*
2013.0-2023.0 APC = -12.28*
* Indicates that the Annual Percent Change (APC) is significantly different from zero at the alpha = 0.05 level.
Final Selected Model: 1 Joinpoint.

I) 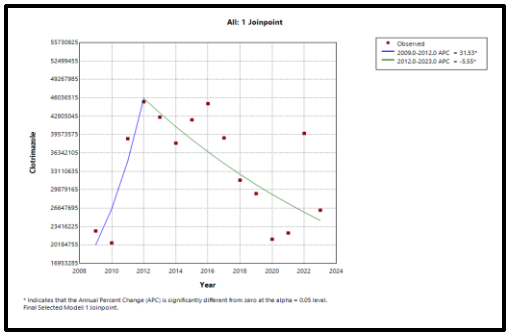
All: 1 Joinpoint
Clotrimazole
55730925
52499455
49267985
46036515
42805045
39573575
36342105
33110635
29879165
26647695
23416225
20184755
16953285
2008 2010 2012 2014 2016 2018 2020 2022 2024
Year
Observed
2009.0-2012.0 APC = 31.53*
2012.0-2023.0 APC = -5.55*
* Indicates that the Annual Percent Change (APC) is significantly different from zero at the alpha = 0.05 level.
Final Selected Model: 1 Joinpoint.

J) 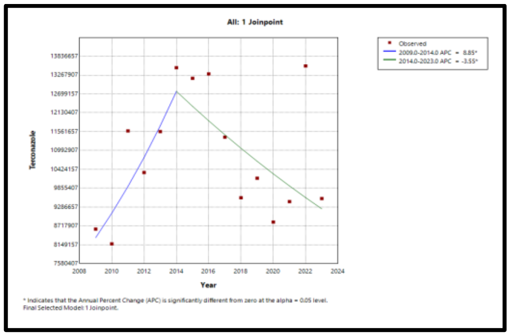
All: 1 Joinpoint
Terconazole
13836657
13267907
12699157
12130407
11561657
10992907
10424157
9855407
9286657
8717907
8149157
7580407
2008 2010 2012 2014 2016 2018 2020 2022 2024
Year
Observed
2009.0-2014.0 APC = 8.85*
2014.0-2023.0 APC = -3.55*
* Indicates that the Annual Percent Change (APC) is significantly different from zero at the alpha = 0.05 level.
Final Selected Model: 1 Joinpoint.

K) 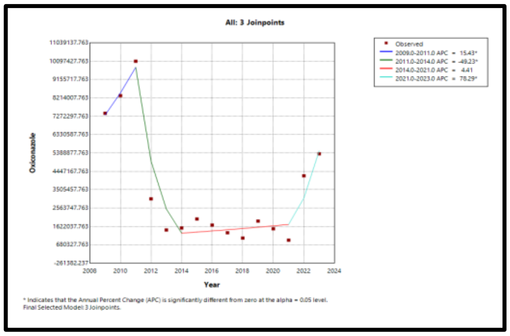
All: 3 Joinpoints
Oxiconazole
11039137.763
10097427.763
9155717.763
8214007.763
7272297.763
6330587.763
5388877.763
4447167.763
3505457.763
2563747.763
1622037.763
680327.763
-261382.237
2008 2010 2012 2014 2016 2018 2020 2022 2024
Year
Observed
2009.0-2011.0 APC = 15.43*
2011.0-2014.0 APC = -49.23*
2014.0-2021.0 APC = 4.41
2021.0-2023.0 APC = 78.29*
* Indicates that the Annual Percent Change (APC) is significantly different from zero at the alpha = 0.05 level.
Final Selected Model: 3 Joinpoints.

L) 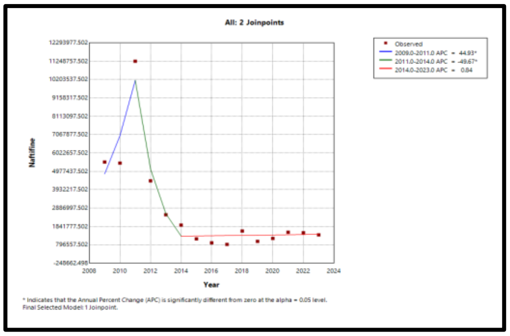
All: 2 Joinpoints
Naftifine
12293977.502
11248757.502
10203537.502
9158317.502
8113097.502
7067877.502
6022657.502
4977437.502
3932217.502
2886997.502
1841777.502
796557.502
-248662.498
2008 2010 2012 2014 2016 2018 2020 2022 2024
Year
Observed
2009.0-2011.0 APC = 44.93*
2011.0-2014.0 APC = -49.67*
2014.0-2023.0 APC = 0.84
* Indicates that the Annual Percent Change (APC) is significantly different from zero at the alpha = 0.05 level.
Final Selected Model: 1 Joinpoint.

M) 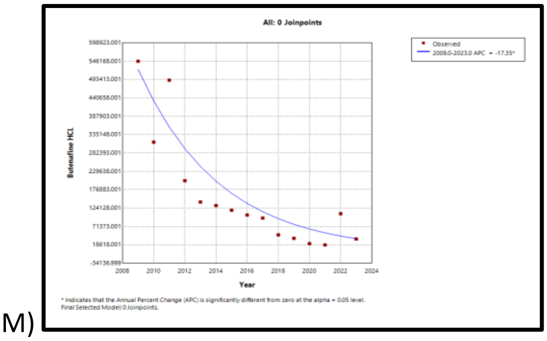

N) 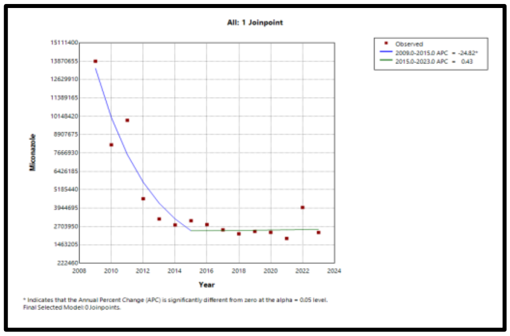

O) 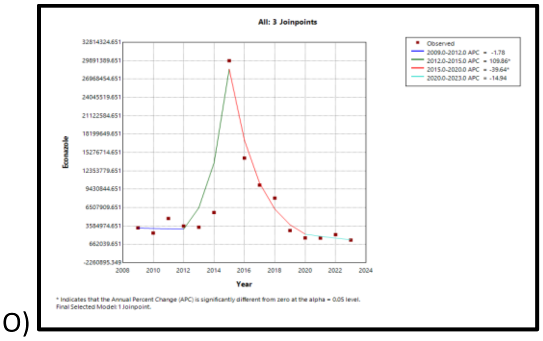

P) 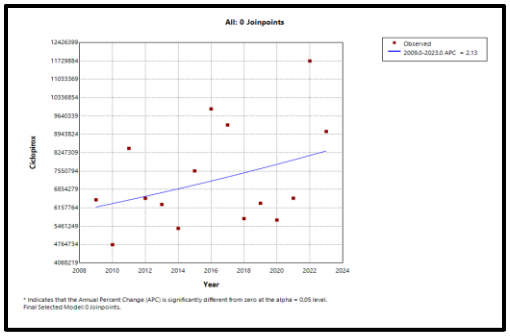

Figure S5. Joinpoint regression for SFIs' medications' spending. (A): Griseofulvin, (B): Tioconazole, (C): Natamycin, (D): Ketoconazole, (E): Terbinafine, (F): Butoconazole, (G): Efinaconazole, (H): Nystatin, (I): Clotrimazole, (J): Terconazole, (K): Oxiconazole, (L): Naftifine, (M): Butenafine, (N): Miconazole (O): Econazole, and (P): Ciclopirox.

A) 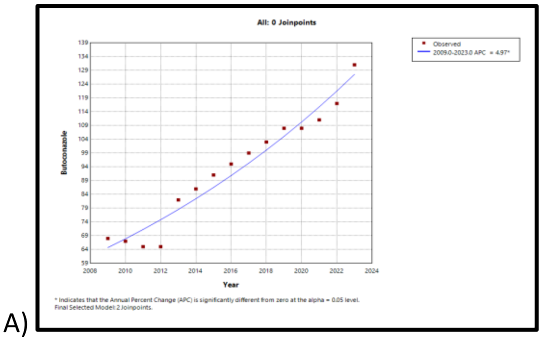

B) 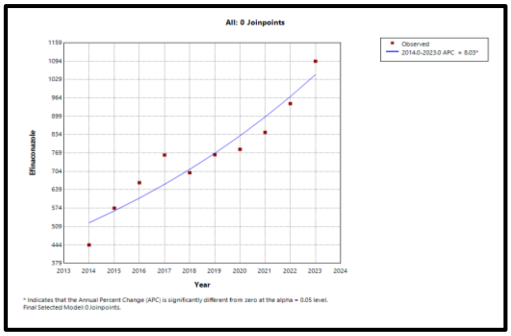

C) 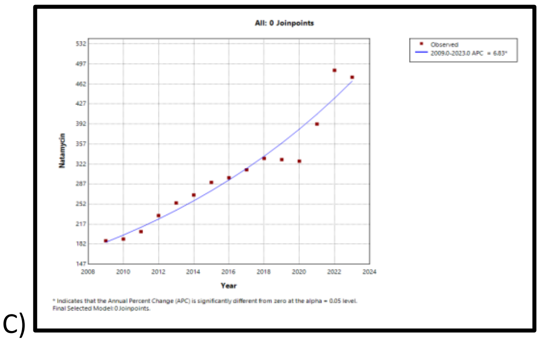

D) 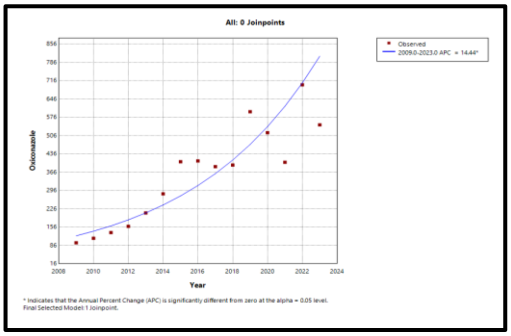

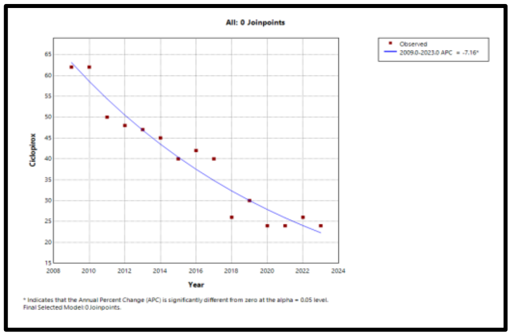
All: 0 Joinpoints
Observed
2009.0-2023.0 APC = -7.16*
Ciclopirox
Year
* Indicates that the Annual Percent Change (APC) is significantly different from zero at the alpha = 0.05 level.
Final Selected Model: 0 Joinpoints.
E)

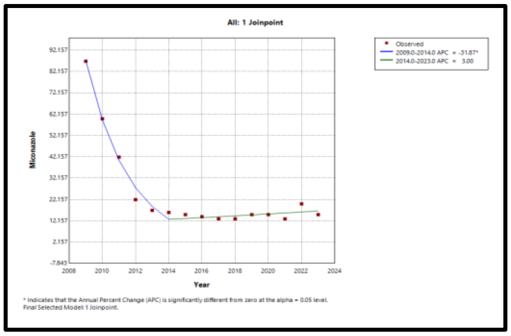
All: 1 Joinpoint
Observed
2009.0-2014.0 APC = -31.87*
2014.0-2023.0 APC = 3.00
Miconazole
Year
* Indicates that the Annual Percent Change (APC) is significantly different from zero at the alpha = 0.05 level.
Final Selected Model: 1 Joinpoint.
F)

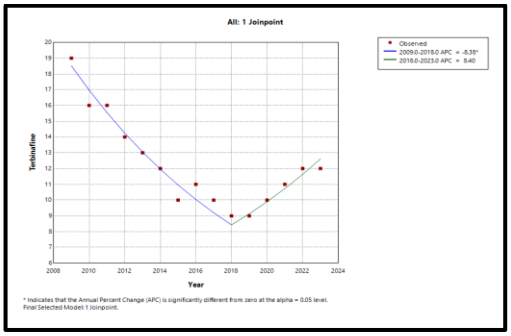
All: 1 Joinpoint
Observed
2009.0-2018.0 APC = -8.38*
2018.0-2023.0 APC = 8.40
Terbinafine
Year
* Indicates that the Annual Percent Change (APC) is significantly different from zero at the alpha = 0.05 level.
Final Selected Model: 1 Joinpoint.
G)

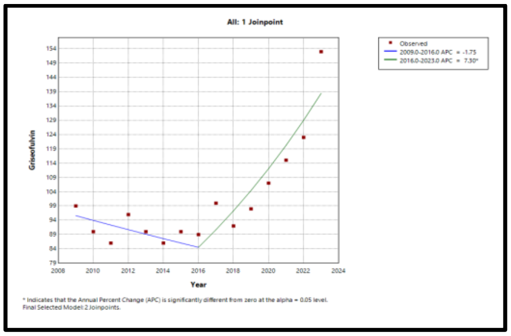
All: 1 Joinpoint
Observed
2009.0-2016.0 APC = -1.75
2016.0-2023.0 APC = 7.30*
Griseofulvin
Year
* Indicates that the Annual Percent Change (APC) is significantly different from zero at the alpha = 0.05 level.
Final Selected Model: 2 Joinpoints.
H)

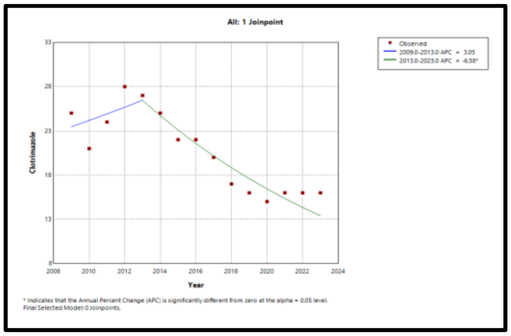
All: 1 Joinpoint
Observed
2009.0-2013.0 APC = 3.05
2013.0-2023.0 APC = -6.58*
Clotrimazole
Year
* Indicates that the Annual Percent Change (APC) is significantly different from zero at the alpha = 0.05 level.
Final Selected Model: 0 Joinpoints.
I)

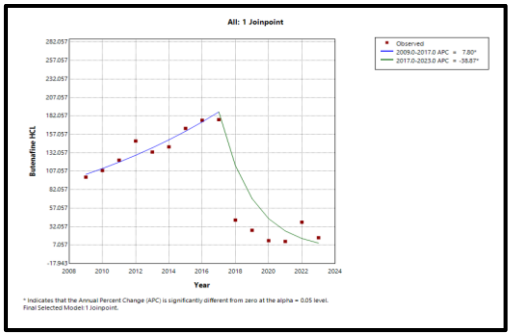
All: 1 Joinpoint
Observed
2009.0-2017.0 APC = 7.80*
2017.0-2023.0 APC = -38.87*
Butenafine HCL
Year
* Indicates that the Annual Percent Change (APC) is significantly different from zero at the alpha = 0.05 level.
Final Selected Model: 1 Joinpoint.
J)

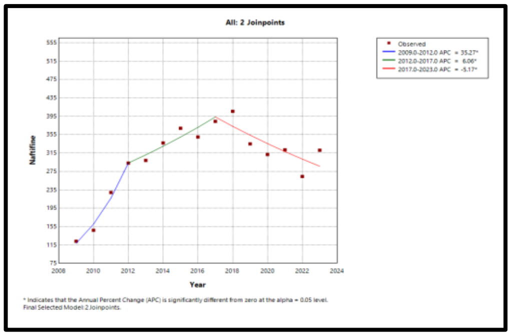
All: 2 Joinpoints
Observed
2009.0-2012.0 APC = 35.27*
2012.0-2017.0 APC = 6.06*
2017.0-2023.0 APC = -5.17*
Naftifine
Year
* Indicates that the Annual Percent Change (APC) is significantly different from zero at the alpha = 0.05 level.
Final Selected Model: 2 Joinpoints.
K)

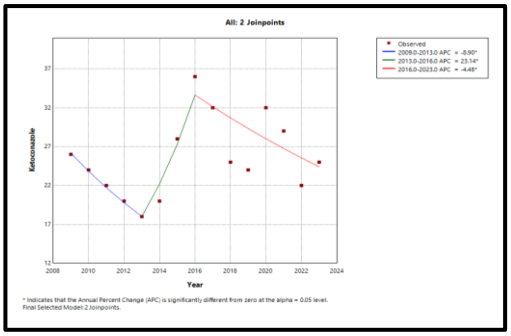
All: 2 Joinpoints
Observed
2009.0-2013.0 APC = -8.90*
2013.0-2016.0 APC = 23.14*
2016.0-2023.0 APC = -4.48*
Ketoconazole
Year
* Indicates that the Annual Percent Change (APC) is significantly different from zero at the alpha = 0.05 level.
Final Selected Model: 2 Joinpoints.
L)

M)
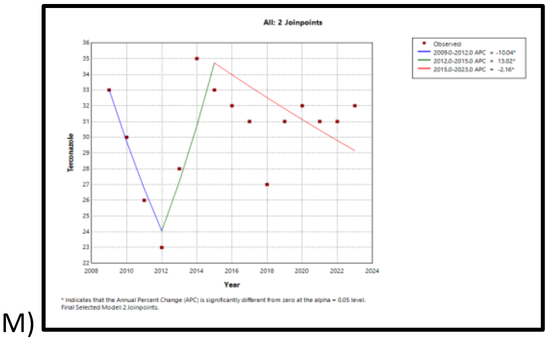

N)
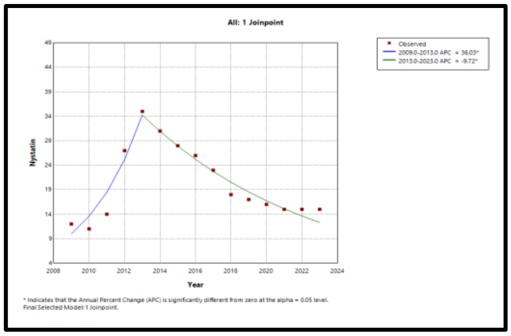

O)
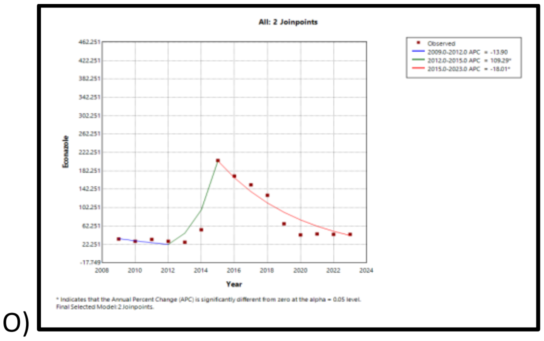

P)
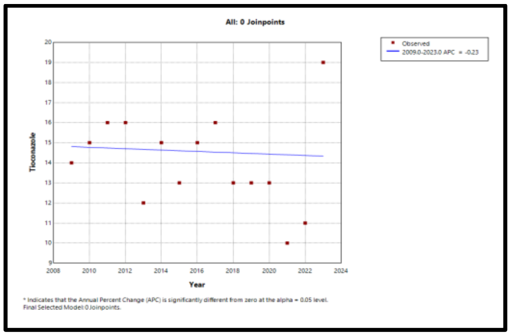

Figure S6. Joinpoint regression for SFIs' medications' price (A): Butoconazole, (B): Efinaconazole, (C): Natamycin, (D): Oxiconazole, (E): Ciclopirox, (F): Miconazole, (G): Terbinafine, (H): Griseofulvin, (I): Clotrimazole, (J): Butenafine, (K): Naftifine, (L): Ketoconazole, (M): Terconazole, (N): Nystatin, (O): Econazole, (P): Tioconazole.

A)
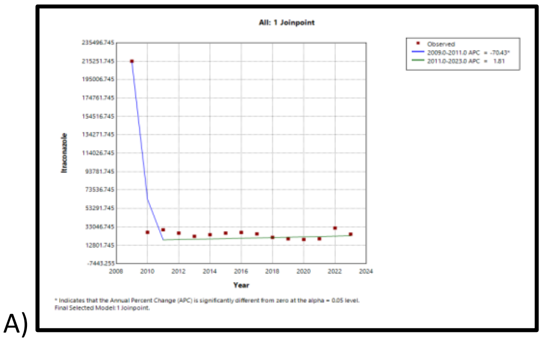

B)
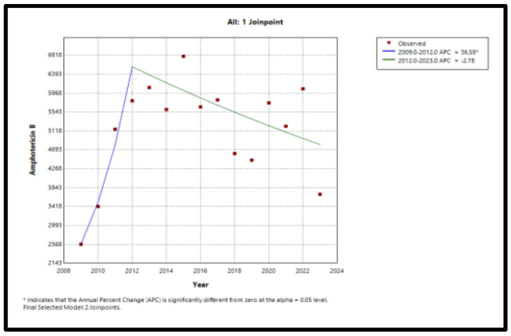

C)
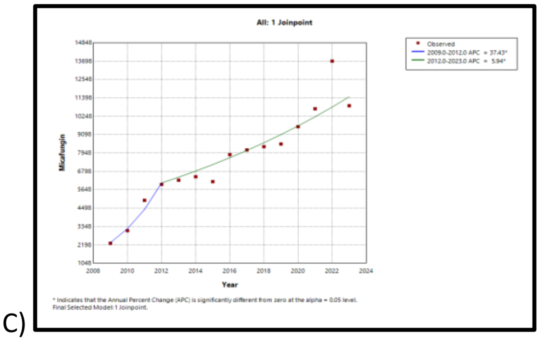

D)
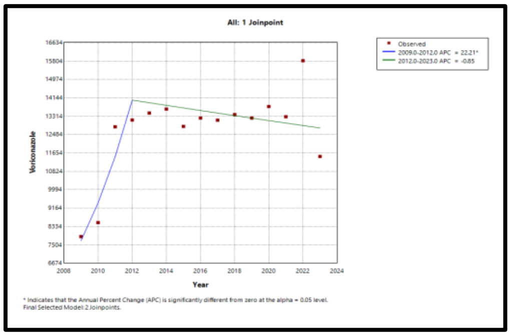

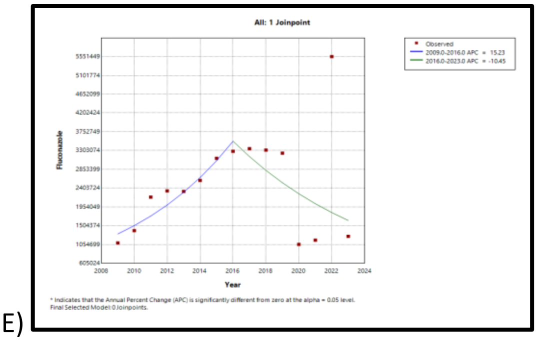

E)

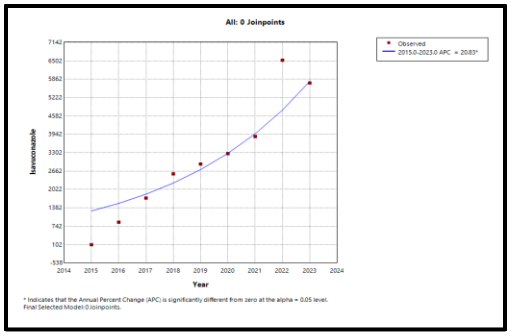

F)

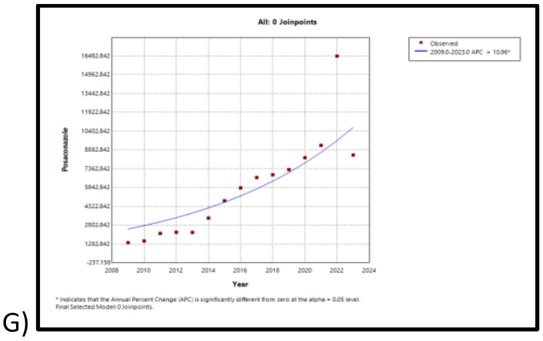

G)

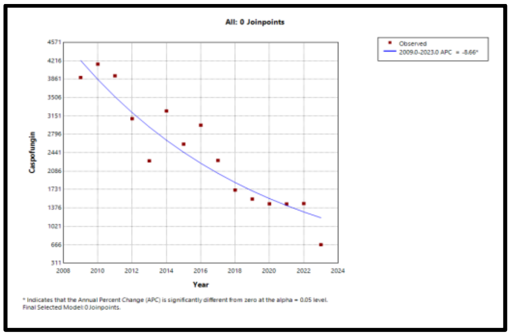

H)

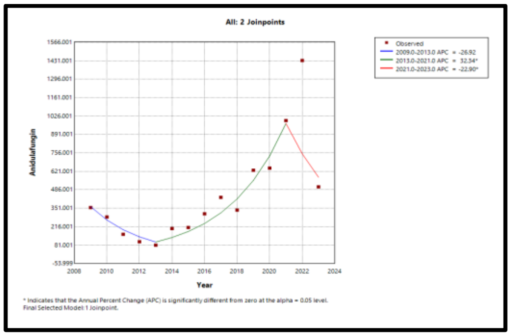

I)

Figure S7. Joinpoint regression for IFI's medications' utilization. (A): Itraconazole, (B): Amphotericin B, (C): Micafungin, (D): Voriconazole, (E): Fluconazole, (F): Isavuconazole, (G): Posaconazole, (H): Caspofungin, (I): Anidulafungin.

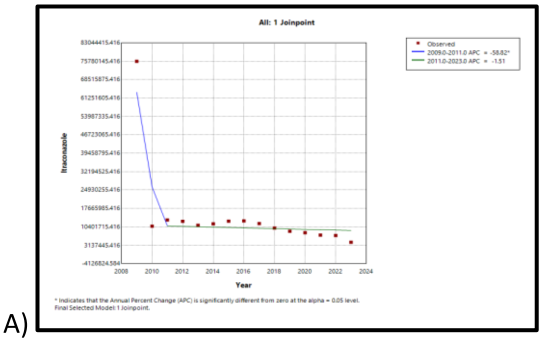

A)

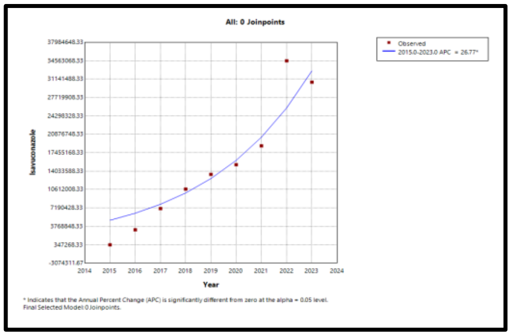

B)

C) D)

E) F)

G) H)

I)

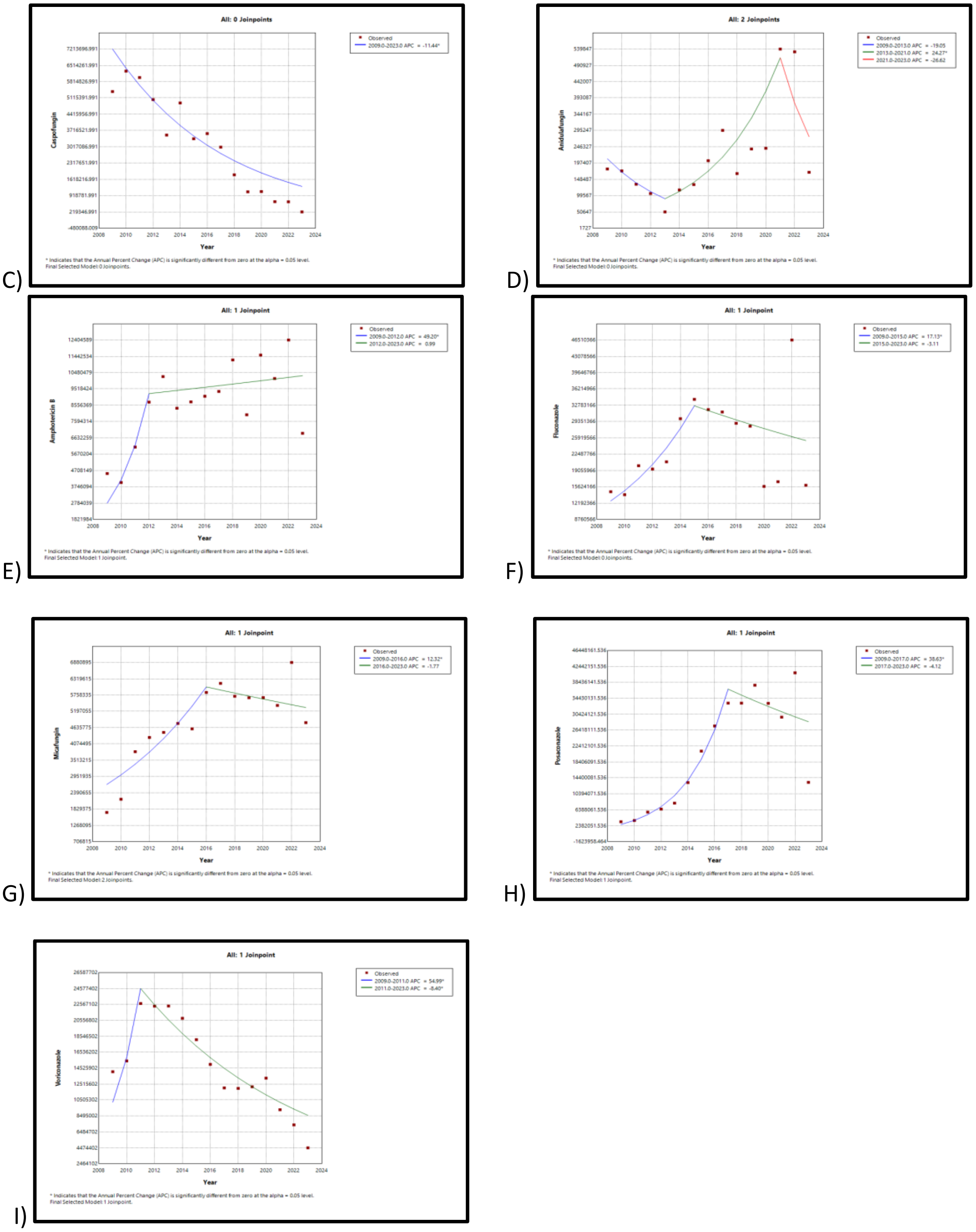

Figure S8. Joinpoint regression for IFIs' medications' spending. (A): Itraconazole, (B): Isavuconazole, (c): Caspofungin, (D): Anidulafungin, (E): Amphotericin B, (F): Fluconazole, (G): Micafungin, (H): Posaconazole and (I): Voriconazole.

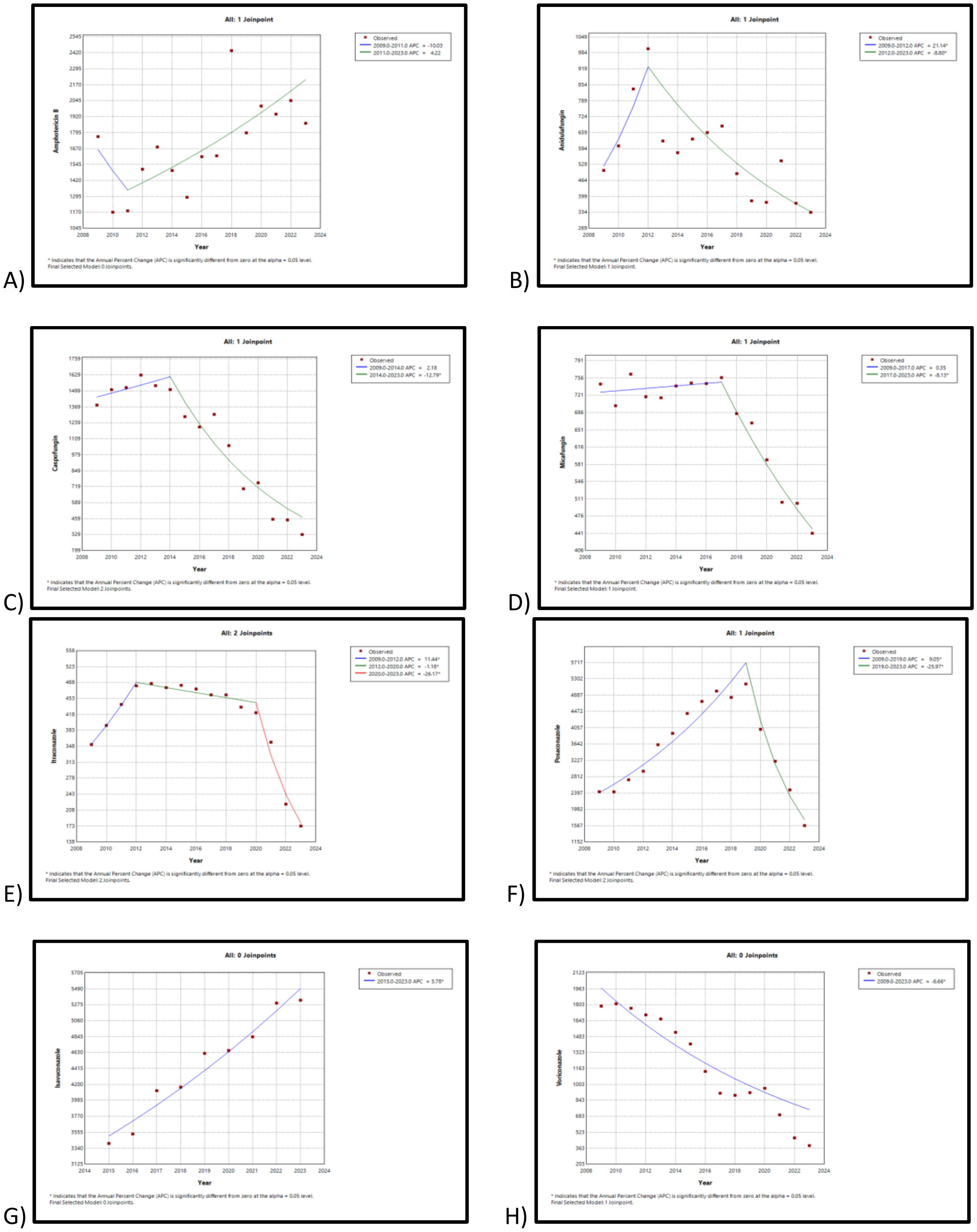

Figure S9. Joinpoint regression for IFIs' medications' prices. (A): Amphotericin B, (B): Anidulafungin, (C): Caspofungin, (D): Micafungin, (E): Itraconazole, (F): Posaconazole, (G): Isavuconazole, (H): Voriconazole.
